# Ivabradine induces RAD51 degradation, potentiating PARP inhibitor efficacy in non-germline *BRCA* pathogenic variant triple-negative breast cancer

**DOI:** 10.1186/s12967-025-06902-8

**Published:** 2025-08-05

**Authors:** Ho Tsoi, George Man Hong Leung, Ellen Pui Sum Man, Chan Ping You, Koei Ho Lam Cheung, Kelvin Yuen Kwong Chan, Chun Gong, Michael Shing Yan Huen, Ui Soon Khoo

**Affiliations:** 1 Department of Pathology, School of Clinical Medicine, Li Ka Shing Faculty of Medicine, The University of Hong Kong, Pokfulam, Hong Kong SAR, China; 2 School of Science and Technology, Hong Kong Metropolitan University, Ho Man Tin, Hong Kong SAR China; 3 Department of Haematology, University of Cambridge, Cambridge, UK; 4 School of Biomedical Sciences, Li Ka Shing Faculty of Medicine, The University of Hong Kong, Pokfulam, Hong Kong SAR China

**Keywords:** Breast cancer, BRCAness, Ivabradine, Olaparib, Synthetic lethality, Targeted therapy, TNBC

## Abstract

**Background:**

Triple-negative breast cancer (TNBC) is an aggressive subtype lacking targetable proteins for treatment. PARP inhibitors (PARPi) are effective in *BRCA*-mutated cancers but have limited utility in non-germline BRCA-mutated (non-g*BRCA*m) TNBC. We hypothesized that inducing BRCAness by targeting RAD51, a key homologous recombination protein, could sensitize non-g*BRCA*m TNBC to PARPi.

**Methods:**

EGFP-tagged RAD51 was generated and EGFP signal was monitored for identifying agents that affected RAD51 protein expression and stability. Cell viability was assayed using cell counting kit-8. Synergism of ivabradine and olaparib was determined using SynergyFinder 3.0. DR-GFP, EJ5-GFP and comet assays were employed to evaluate the degree of DNA repair and damage, respectively. Protein and mRNA levels were determined by western blot and qPCR, respectively. ChIP was used to determine the binding to ATF6 to the promoter of FBXO24. CoIP was employed to determine the interaction between RAD51 and FBXO24. Xenografts on nude mice and PDTX were in vivo models for validating the combined effect of ivabradine and olaparib.

**Results:**

Using an EGFP-RAD51 reporter, we identified ivabradine as a RAD51-reducing agent. In vitro studies with TNBC cell lines demonstrated that ivabradine synergized with PARPi to reduce cell viability (ZIP score > 10), induce apoptosis, and impair HR-mediated DNA repair. This synergy was confirmed in vivo using xenografts and patient-derived tumor xenografts, where co-treatment with clinical grade ivabradine (Coralan) and PARPi olaparib (Lynparza) led to substantial tumor growth inhibition without notable toxicity. Mechanistically, ivabradine triggered ER stress, activating ATF6 to upregulate FBXO24-dependent ubiquitination, leading to RAD51 degradation, resulting in the condition of BRCAness. Chromatin immunoprecipitation and co-immunoprecipitation confirmed the ATF6-FBXO24-RAD51 cascade. These findings reveal a novel mechanism by which ivabradine, an FDA-approved cardiac drug, induces BRCAness, by degrading RAD51 via the ATF6-FBXO24 axis, thus, by mimicking HR deficiency hypersensitizes BRCA-proficient TNBC to olaparib.

**Conclusion:**

This study highlights the translational potential of repurposing ivabradine as a therapeutic strategy for non-g*BRCA*m TNBC. By addressing a critical unmet need of this aggressive breast cancer subtype, it can potentially expand the utility of PARPi.

**Graphical abstract:**

Schematic diagram illustrates the synergistic effect of IVA and OLA. IVA treatment results in enhanced ER stress, leading to the activation of ATF6. The activated ATF6 translocates to the nucleus and binds to the promoter of FBXO24 to induce its expression. FBXO24 mediates RAD51 protein degradation via the ubiquitin-proteasome system. The reduction of RAD51 expression contributes to the feature of BRCAness. Adding PARP inhibitor OLA can prevent single-strand DNA damage from repairing, subsequently becoming DNA double-strand breakage (DSB). The low expression of RAD51 mediated by IVA compromises the mechanism for repairing DSB via HR, leading to the accumulation of DSB. Eventually, cell death is induced.

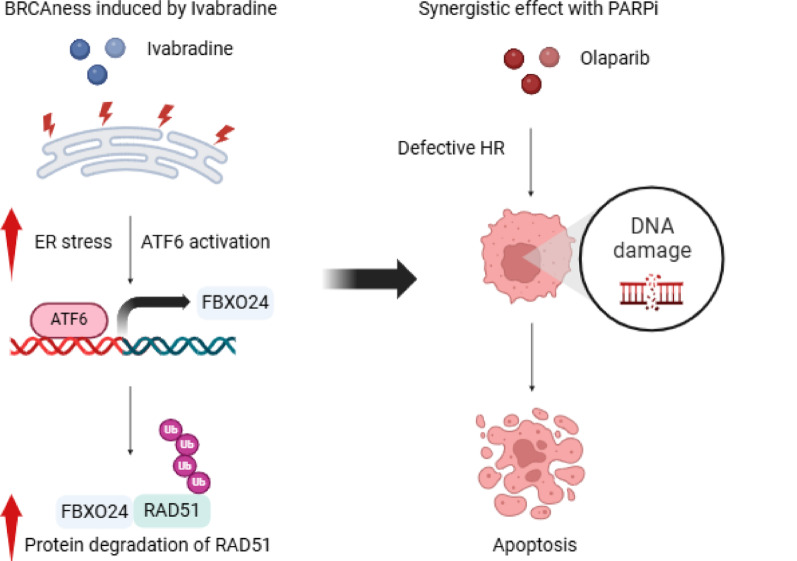

**Supplementary Information:**

The online version contains supplementary material available at 10.1186/s12967-025-06902-8.

## Background

Triple-negative breast cancer (TNBC), accounting for 15% of invasive breast cancers, is characterized by the absence of estrogen receptor, progesterone receptor, and human epidermal growth factor receptor 2. This subtype exhibits aggressive behavior, early metastasis, and poor survival outcomes. Chemotherapy remains the primary treatment, but resistance often develops. Targeted therapies like CDK4/6 or PI3K inhibitors show limited efficacy, whilst neoadjuvant immune checkpoint inhibitor, combined with chemotherapy is a recent advance. PARP inhibitors (PARPi) have revolutionized cancer therapy by exploiting the concept of synthetic lethality in cancers with homologous recombination deficiency (HRD) due to germline *BRCA*-mutation (g*BRCA*m) [1, 2]. PARPi, through the interference of single-strand breast repair enzyme PARP, results in the accumulation of unrepaired DSBs that leads to cell death of cancer cells deficient in BRCA1/2 proteins. However, the application of PARPi in wt*BRCA* TNBC has been less successful, since intact homologous recombination (HR) pathways can repair DNA damage caused by PARPi. Therefore, inducing'BRCAness', a state mimicking *BRCA* mutations, in these cancers could broaden the therapeutic window of PARPi.

HR is a high-fidelity DNA repair pathway critical for repairing DNA double-strand breaks (DSBs), BRCA1 and BRCA2 being two key players for this [3]. In *BRCA1/2*-deficient tumors, HR is impaired, leading to reliance on error-prone repair mechanisms and sensitivity to PARPi [4]. RAD51, a central protein in HR, facilitates the search for homologous sequences and strand pairing during repair, ensuring genomic stability [5, 6]. Targeting RAD51 to induce HR deficiency, i.e. BRCAness, would represent a promising strategy to expand PARPi efficacy to non-gBRCAm TNBC.

ER stress has been reported to suppress HR by enhancing the degradation of RAD51 [7]. RAD51, a key mediator of HR, is a protein essential for repairing damaged DNA DSBs through HR [5, 6]. RAD51 mutations are rare, and its functional expression in most breast cancers makes it an attractive therapeutic target. We identified ivabradine (IVA), an FDA-approved hyperpolarization-activated cyclic nucleotide-gated (HCN) channel blocker, as a potent inducer of RAD51 degradation. HCN channels, particularly HCN2 and HCN3, are overexpressed in breast cancer and associated with poor survival outcomes [8]. IVA, originally used to treat chronic angina, has been shown to induce ER stress in cancer cells [8].

In this study, we demonstrated that IVA triggered ER stress, activating the ATF6-FBXO24 axis to ubiquitinate and degrade RAD51, thereby impairing HR and inducing BRCAness. This mechanism hypersensitizes HR-proficient TNBC to PARPi, creating a synthetic lethal interaction. By repurposing IVA to mediate RAD51 down-regulation, we provide a novel therapeutic strategy for non-g*BRCA*m TNBC, addressing a critical unmet need in this aggressive subtype.

## Methods

### Cell culture, molecular cloning, and transfection

Human breast cancer cell lines estrogen receptor (ER + ve) cell MCF-7, wild-type *BRCA* TNBC cells MDA-MB-231 and MDA-MB-453, homozygous *BRCA1* deletion cell MDA-MB-436, as well as heterozygous *BRCA2* mutant cell MDA-MB-468 were purchased from ATCC. The HEK293 human kidney cell was obtained from Thermofisher. Cell lines were cultured and maintained were cultured and maintained in Dulbecco's Modified Eagle Medium (DMEM; 12800082, Gibco) supplemented with 10% FBS (A5256801, Gibco) and 1% penicillin/streptomycin (15140122, Gibco). MCF-10A, a non-neoplastic epithelial breast cell line, was obtained from ATCC and cultured in DMEM/F12 with 5% horse serum (16050–122, Gibco), 20 ng/mL of EGF (PHG0311, Gibco), 0.5 mg/mL of hydrocortisone (H0888, Sigma), 100 ng/mL of cholera toxin (C8052, Sigma), 10 μg/mL of insulin (12585014, Gibco). Human *RAD51* was cloned into *pcDNA3.1* with myc tagged. cDNA from HEK293T was used. *RAD51* was amplified with primers: BamH1_myc_RAD51-F (5'-CTG ACA GGA TCC ATG GAG CAG AAA CTC ATC TCT GAA GAG GAT CTG ATG GCA ATG CAG ATG CAG C-3') and XbaI_stop_RAD51-R (5'-TGA CTG TCT AGA TCA TCA GTC TTT GGC ATC TCC CAC-3') using Platinum™ SuperFi II DNA Polymerase (12361010, Invitrogen). The amplicon and *pcDNA3.1* were digested with *BamH*I (R0136S, NEB) and *Xba*I (R0145S, New England Biolabs). The digested DNA products were ligated using T4 DNA ligase (M0202S, New England Biolabs) to generate *pcDNA3.1_myc_RAD51*. Human RAD51 was cloned into *pEGFPN1*. *RAD51* was amplified with primers: Pst1_RAD51-F (5'- CGC TGC AGA TGG CAA TGC AGA TGC AG-3') and BamH1_stop_RAD51-R (5'- CGG GAT CCC CGT CTT TGG CAT CTC CCA C-3') using Platinum™ SuperFi II DNA Polymerase (12361010, Invitrogen). The amplicon and *pEGFPN1* were digested with *Pst*1 (R0140S, New England Biolabs) and *BamH*1 (R0136S, New England Biolabs). The digested DNA products were ligated using T4 DNA ligase (M0202S, New England Biolabs) to generate *pEGFP_RAD51*. According to the manufacturer's manual, transfection was performed using Lipofectamine 2000 (11668027; Invitrogen) or Lipomaster 3000 (TL301; Vazyme).

### Cell viability assay and synergistic effect

5000–10,000 were seeded in 96-well plate. Triplicate samples were used. Absorbance at 450 nm was recorded using microplate reader Infinite F200 (Tecan Trading AG). Cell counting kit 8 (CCK-8; C0038, Beyotime Biotech. Inc.) was employed. All inhibitors used was listed in Table S1. Synergistic effect was detected using SynergyFinder 3.0 using four-parameter logistic regression (LL4) for curve-fitting [9]. The synergistic effect was determined using ZIP synergy model. When a ZIP score exceeded 10, the interaction between the two drugs was synergistic. IC50 value was determined using Prism8 (GraphPad) with log(inhibitor) vs. response robust fit model.

### Western blot

Cells were lysed using lysis buffer (Tris–Cl, pH7.4, 150 mM NaCl, 0.5% NP-40, 1% Triton X-100, 0.1% SDS). 50 mg of tumor tissues from xenografts were homogenized in 500 μL of lysis buffer. Protein concentration was determined using DC protein assay (5000112, BioRad). 20 μg of proteins were analyzed using SDS-PAGE. The proteins were transferred to the PVDF membrane (1620177, BioRad). 5% non-fat milk or 5% BSA in TBST was used to block the membrane. The membrane was incubated with primary antibody at 4 °C overnight. After washing with TBST, the membrane was incubated with a secondary antibody for 2 h at room temperature. A signal was developed using BeyoECL Star (P0018AM, Beyotime Biotech. Inc.) or BeyoECL Moon (P0018FM, Beyotime Biotech. Inc.). The following primary antibodies were used: anti-RAD51 (1:1,000; #8875, Cell Signaling Technology), anti-BRCA1 (1:1,000; #9010, Cell Signaling Technology), anti-BRCA2 (1:2,000; #10741, Cell Signaling Technology), anti-HCN2 (APC-030; Alomone Labs), anti-HCN3 (APC-057; Alomone Labs), anti-GFP (1:5000; 632380; Clontech), anti-HSP90 (1:5000; #4878, Cell Signaling Technology), anti-GAPDH (1:20,000; sc-47724, Santa Cruz Biotechnology), anti-phos-ATM (Ser1981) (1:1000; #5883, Cell Signaling Technology), anti-γH2AX (Ser139) (1:1000; #9718, Cell Signaling Technology), anti-cleaved caspase 3 (1:1,000; #9664, Cell Signaling Technology), anti-FBXO24 (1:,2000; PA5-97737, ThermoFisher Scientific), anti-AIF (1:1,000; #4642, Cell Signaling Technology); anti-β-tubulin (1:10,000; #2146, Cell Signaling Technology) anti-MYC tag (1:4,000; #2276, Cell Signaling Technology), anti-ubiquitin (1:5000; sc-166553, Santa Cruz Biotechnology), and anti-K48-linked polyubiquitin (1:2000; #4289, Cell Signaling Technology). Secondary antibodies, goat anti-mouse (1:5000; 31430, ThermoFisher Scientific) and goat anti-rabbit (1:4000; 31460, ThermoFisher Scientific), were used. ECL signals were recorded using GE Amersham Imager AI680 (GE HealthCare) or Alliance Q9 (Uvitec Ltd.). Representative images were shown. Band intensity was determined using Image J [10].

### Functional assays

Cignal ERSE Reporter Kit Reporter Kit (CCS‐2032L; Qiagen) was employed to determine ER stress. Chemiluminescence was recorded by microplate reader Infinite F200 (Tecan Trading AG). Comet assay was performed using CometAssay Single Cell Gel Electrophoresis Assay kit (4250–050-K, Biotechne). The alkaline condition was used. Images were observed and recorded using a H600L (Nikon) microscope with DS-Qi2 (Nikon). %DNA in head and tail were determined using ImageJ with OpenComet v1.3.1 plugin, and 200 nuclei were analyzed. GreenNuc™ Caspase-3 Assay Kit (C1168, Beyotime Biotech. Inc.) was used. Cells (20,000) were seeded in 96-well plates. Triplicate samples were used. GFP signal was recorded using a microplate reader Infinite F200 (Tecan Trading AG). TUNEL assay was performed using TUNEL BrightGreen Apoptosis Detection Kit (A112091, Vazyme). The cells were analyzed using a flow cytometer BD FASCFortessa (BD Biosciences). Cells were gated with 1) FSC-A and SSC-A, 2) FSC-A and FSC-H for singlet. Alexa488 signal was recorded. Cells in Q3 were regarded as TUNEL-positive cells.

### Tracking EGFP-RAD51 signal

1 × 10^6^ cells of HEK293, MCF-7, MDA-MB-231, MDA-MB-436, MDA-MB-453 and MDA-MB-468 cells were seeded and transfected with 2 µg of pEGFP-RAD51 using Lipofectamine 2000 (11668027; Invitrogen). 24 h posttransfection, 1 × 10^5^ cells were placed in 24 well plates. Triplicate samples were used. 5 µM of IVA was added to treat the cells for 48 h. Then, the medium was replaced with fresh and serum-free DMEM without phenol red (21063029; ThermoFisher). EGFP signal at the endpoint was captured using a microplate reader Infinite F200 (Tecan Trading AG).

For tracking the EGFP signal continuously, 1 × 10^6^ cells of MDA-MB-231 and MDA-MB-453 in 6-well plates were transfected with 2 µg of Lipomaster 3000 (TL301; Vazyme). 24 h post-transfection, 1 × 10^5^ cells were placed in 24 well plates. Triplicate samples were used. Prior to IVA treatment, the transfected cells in 24 well plates were treated with 10 µg/mL of cycloheximide (CHX) to inhibit protein synthesis for 6 h. Then, the medium was replaced with fresh and serum-free DMEM without phenol red (21063029; ThermoFisher), which contained 5 µM of IVA and 10 µg/mL of CHX. After 2 h of incubation, the cells were excited each minute using a laser at 488 nm, and the EGFP signal was captured using MD SpectraMax i3x fluorescent microplate reader (Molecular Devices).

### HR and NHEJ activity assays

*pDRGFP* (Addgene plasmid #26475) [11] and *pCBASceI* (Addgene plasmid #26477) [12] were gifts from Maria Jasin. pimEJ5GFP (Addgene plasmid Plasmid #44026) [13] was a gift from Jeremy Stark. 1 × 10^6^ cells in a 6-well plate were transfected with 1 μg of *pDRGFP* or *pimEJ5GFP* and 1 μg of *pCBASceI*. At 48 h post-transfection, cells were harvested and analyzed using a flow cytometer BD FASCFortessa (BD Biosciences). Results were analyzed using FlowJo v10 (BD Biosciences). Cells were gated with 1) FSC-A and SSC-A, 2) FSC-A and FSC-H for singlet. GFP signal was recorded. Mean Fluorescence Intensity (MFI) was determined. MDA-MB-231, MDA-MB-436, MDA-MB-453, and MDA-MB-468 (1 × 10^5^ cells) were transfected with 0.5 μg of *pDRGFP* or *pimEJ5GFP* and 0.5 μg of *pCBASceI* in a 24-well plate. Triplicate samples were used. At 48 h post-transfection, the GFP signal was recorded using a microplate reader Infinite F200 (Tecan Trading AG).

### Chromatin immunoprecipitation (ChIP)

ChIP was performed using Pierce™ Magnetic ChIP Kit (25,157, ThermoFisher Scientific) according to the manufacturer's instructions. 2 × 10^6^ cells were used. Cells were fixed with 4% formaldehyde. DNA was fragmented using Sonifier SFX250 (50% power, 30 s on and 1 min off cycle for 5 min; Branson Ultrasonics) with microtip (9655V15). Sonication was performed on ice. qPCR was performed with the following primers: FBXO24 − 319 to − 208-F (5′-GGA GGG GAA CAA GGA TAG AGC-3′), FBXO24 -319 to -208-R (5′-TGT CCC CTC CTC TTG GTT GG-3′), FBXO24 − 688 to − 493-F (5′-CGC CGG TTA GAC AGG TTC A-3′), FBXO24 − 688 to − 493-R (5′-GGT CCC GTC AGT CAG GCA-3′), FBXO24 − 793 to − 663-F (5′-AAA TGA GGG ACC CGG TTG G-3′) and FBXO24 − 793 to − 663-R (5′-GGG ACC CTG AAC CTG TCT AAC-3′). Relative enrichment was determined according to the previous work [14].

### Co-immunoprecipitaion

MDA-MB-231 and MDA-MB-453 were transfected with 2 μg of *pcDNA3.1_myc_RAD51* using Lipomaster 2000 (TL201-01, Vazyme). At 48 h post-transfection, the cells were lysed in 200 μL of binding buffer (Tris–Cl, pH 7.4, 100 mM NaCl, 0.5% NP-40, 0.5% Triton X-100, 10% glycerol). 90 μL of the lysate was incubated with anti-mouse IgG (1:100; 02–6502, Invitrogen) or anti-myc (1:100; #2276, Cell Signaling Technology) at 4 °C overnight. The immunoprecipitant was incubated with 50 μL of Dynabeads (10001D, Invitrogen) at room temperature for 2 h. The beads were washed with 1 mL of binding buffer. The immunoprecipitant was eluted with 50 μL of 2 × SDS sample buffer. The samples were analyzed through western blots.

### Reverse transcription and qPCR

Total RNA was extracted using Trizol reagent (15596026, Invitrogen). 0.5 μg of RNA was used to synthesize cDNA using High-Capacity cDNA Reverse Transcription Kit (4368814, Applied Biosystems). PowerUp™ SYBR™ Green Master Mix for qPCR (A25741, ThermoFisher) was used. StepOne™ Real-Time PCR System (Applied Biosystems) was used. The following primers were used: RAD51-F (5′-ACC GCC CTT TAC AGA ACA GA), RAD51-R (5′-CCA CTT GAG CTA CCA CCT GA-3′), BRCA1-F (5′-GCA TGC TGA AAC TTC TCA ACC A-3′), BRCA1-R (5′-GTG TCA AGC TGA AAA GCA CAA ATG A-3′), BRCA2-F (5′-AGA CTG TAC TTC AGG GCC GTA CA-3′), BRCA2-R (5′-GGC TGA GAC AGG TGT GGA AAC A-3′), FBH1-F (5′-GGT CCT GAT CCC ATTC CTG A-3′), FBH1-R (5′-GTG CCT CAG GAC CTC ACT AG-3′), FBXO24-F (5'-AAG GAC TTC TTC TGG GAG GC-3′), FBXO24-R (5'-GAC TAG GCT AGC ATG AGG GG-3′), FBXW7-F (5′-GGT GCT GGA CTT TGA TGT GG-3'), FBXW7-R (5′-ATC CTG CAC CAC TGA GAA CA-3′), FBXW11-F (5′-GAC GCT GGG TAG ATG CAA AG-3′), FBXW11-R (5'-TCT ACT TGA AGC CGG GGA AG-3′), GAPDH-F (5′-GCA AAT TCC ATG GCA CCG T-3′), GAPDH-R (5′-TCG CCC CAC TTG ATT TTG G-3′), ACTIN-F (5′-ATC GTG CGT GAC ATT AAG GAG AAG-3′) and ACTIN-R (5′-AGG AAG GAA GGC TGG AAG AGT G-3′). ∆∆CT method was used to determine the relative expression of candidate genes. ACTIN or GAPDH were used as the internal controls.

### Inhibitor screening

Table S1 shows the information on the inhibitors used in the screening. 1 × 10^7^ cells of MDA-MB-231 and MDA-MB-453, known wt*BRCA* TNBC cells were transfected with 10 µg of *pEGFP_RAD51* using Lipomaster 3000 (TL301; Vazyme). 24 h posttransfection, 1 × 10^5^ transfected cells were seeded in a 96-well plate. After 24 h, the cells were treated with 5 µM of each chemical shown in Table S1. EGFP signal and cell viability were recorded and compared with the untreated group after 48 h using Infinite F200 (Tecan Trading AG). A heatmap with four biological replicates from each of the cell lines was plotted.

### Chemicals, drugs, and small interfering RNA (siRNA)

Ivabradine hydrochloride (IVA; SML0281) and 4-PBA (SML0309) were purchased from Sigma and dissolved in DMSO. The PARPi Olaparib (OLA; AZD2281), MG132 (S2619), IRE1α kinase inhibitor (Kira6; S8658), and PERK inhibitor (GSK2606414; S7307) were purchased from Selleckchem and dissolved in DMSO. The PARPi Niraparib (NIRA; HY-10619), ATF6 inhibitor (Ceapin-A7; HY-108434) and Z-VAD-FMK (HY-16658B) were purchased from MedChemExpress and dissolved in DMSO. For in vivo use, clinical grade IVA and OLA, Coralan (COR 5 mg; Servier) and Lynparza (LYN 150 mg; AstraZeneca) were used respectively, and diluted in saline and 5% DMSO in saline, respectively. According to the manufacturer's instructions, cells were transiently transfected with Oligofectamine™ Transfection Reagent (12,252,011, Invitrogen). FBXO24 siRNA (siFBXO24; 135,373, Invitrogen) and the Non-targeting siRNA (siCtrl) Silencer™ Select Negative Control No. 1 siRNA (4,390,843, Invitrogen) were used.

### Xenografts and patient-derived tumor xenografts

The procedures were reviewed and approved by Committee on the Use of Live Animals in Teaching and Research, The University of Hong Kong (5236–19). Female nude mice at 6–8 weeks old were employed. 1 × 10^7^ of MDA-MB-231 or MDA-MB-453 cells were inoculated onto the mammary fatpad of the mice. The mice were randomly assigned into different groups when the tumors were visible. The mice were treated with 1 mg/Kg of COR and/or 25 mg/Kg of LYN via subcutaneous injection. The treatment frequency was twice per week. Approval was granted from The Institutional Review Board of the University of Hong Kong/Hospital Authority Hong Kong West Cluster (HKU/HA HKW IRB No. UW 16–391) for collecting human tissue and blood samples. Serum samples from triple-negative breast cancer cases used to construct PDTX5 and PDTX8 were collected. The DNA was isolated using QIAamp DNA Blood Mini Kit (51,104). Sanger sequencing confirmed no pathogenic germline *BRCA1* and *BRCA2* mutations was found (results, primers and genome coordinates as indicated in Table S3). PDTX5 and PDTX8 were established from a previous study. 2 mg/Kg of COR and/or 124 mg/Kg of LYN were employed. The mice were treated with gavage daily. The physical parameters of the tumors were measured and recorded using an electronic caliper. The tumor volume was calculated using Volume = (W × W × L)/2.

### Targeted DNA sequencing

Genomic DNA from the cell lines were extracted using QIAamp DNA Micro Kit (56304, Qiagen). Germ-line DNA was extracted using QIAamp DNA Blood Mini Kit (51104, Qiagen) from blood samples collected from breast cancer patients (PDTS5 and PDTX8). The procedures were reviewed and approved by Committee on the Use of Live Animals in Teaching and Research, The University of Hong Kong (5236–19). The DNA samples were handled and NGS analysis using TSO500 (20076480, Illumina) was performed by the company PhaseScientific International Limited. The results were shown in Table S4.

### Statistical analysis and schematic diagram

All numerical data were processed in Excel (Microsoft). Statistical analysis was performed using Prism10.1 (GraphPad). Data were expressed as mean ± SD from at least three independent experiments. Two-tailed Students' t-test were performed to compare the variables of the 2 sample groups. One-way ANOVA and two-way ANOVA with Tukey’s Bonferroni’s multiple comparisons test were employed to determine the statistical significance for multiple groups. The heatmap was plotted using Morpheus (https://software.broadinstitute.org/morpheus). The schematic diagram was created using images available in BioRender.

## Results

### IVA reduced RAD51 expression in breast cancer cells

**Fig. 1:**
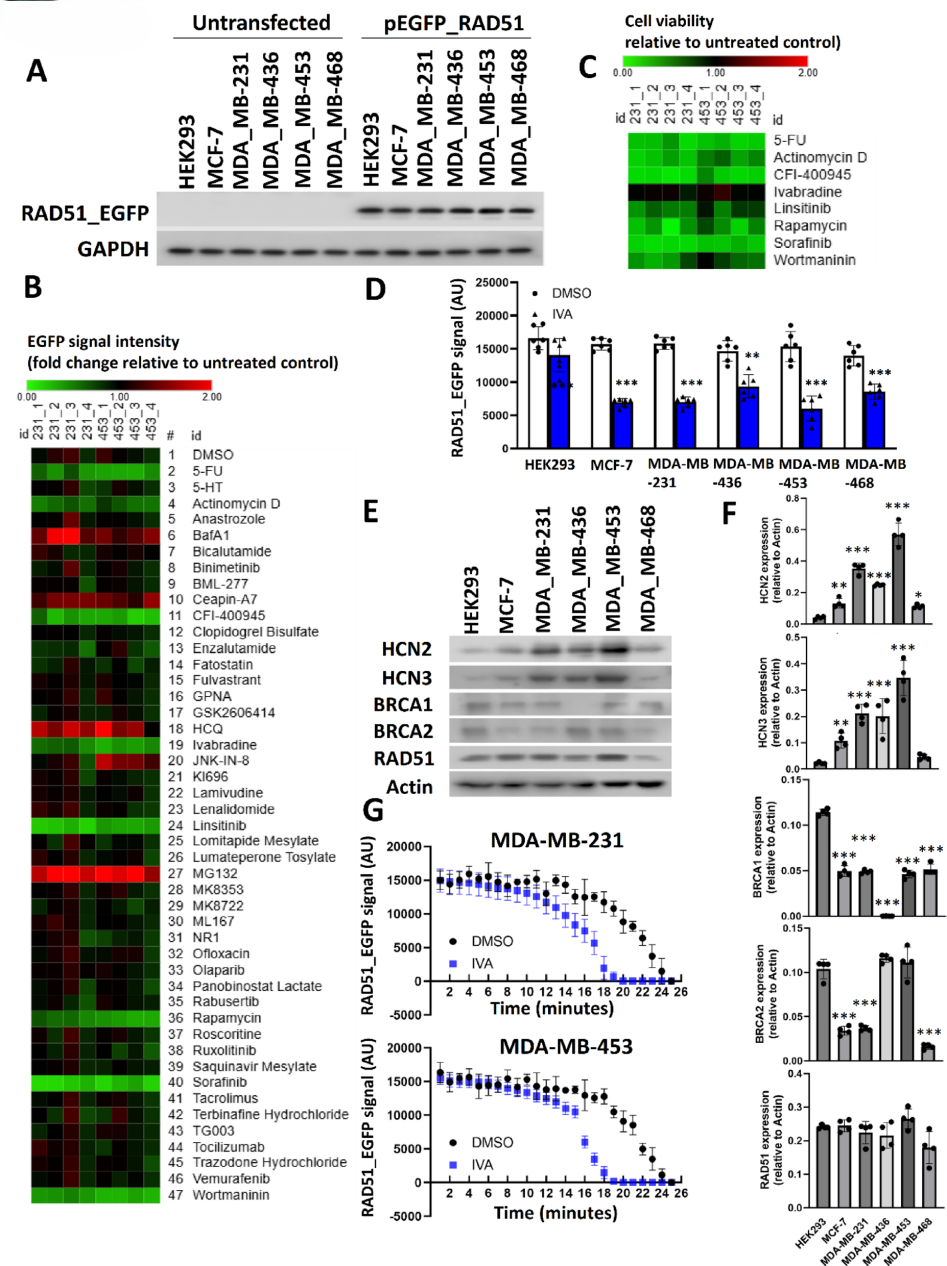
Identification of IVA as a modifier of ectopic EGFP-tagged RAD51. **A** The ectopic expression of RAD51_EGFP in the cell lines. Western blot with anti-EGFP was employed to detect the EGFP-tagged RAD51. GAPDH was the loading control. **B** Screening to identify inhibitors to reduce EGFP-tagged RAD51 in MDA-MB-231 and MDA-MB-453. The cells were treated with 5 µM of each inhibitor for 48 h. The EGFP signal was recorded and compared to the untreated control. A heatmap was plotted to show the effect of the chemicals on the EGFP signal. **C** Heatmap showed the effect of the selected inhibitors on cell viability. CCK8 assay was used after 48 h of the treatment. **D** The treatment of IVA significantly reduced the expression of EGFP-tagged RAD51 in MDA-MB-231 and MDA-MB-453 but not in HEK293. Students’ t-test was used for the statistical analysis. The result was shown as mean ± SD from 6 independent experiments. **E** The expression of BRCA1, BRCA2, RAD51, HCN2 and HCN3. Western blot was employed to examine the expression of the indicated proteins in the cell lines. Actin was used as the loading control. **F** Quantification of E. Image J was employed to determine the band intensity in E. The band intensity of the candidate protein relative to actin was determined. Results showed mean ± SD from 4 independent experiments. **G** The effect of IVA on the protein stability of EGFP-tagged RAD51. After 6 h, the EGFP signal was recorded per minute for 25 min. The result was shown as mean ± SD from 6 independent experiments. ** represents *P* < 0.01. *** represents *P* < 0.001

RAD51, BRCA1, and BRCA2 are essential for homologous recombination, a high-fidelity DNA repair pathway that supports normal cell proliferation and organismal development [15]. We initiated our study by evaluating compounds that could modulate RAD51 expression, crucial for HR. Using an EGFP-RAD51 reporter construct in five breast cancer cell lines (MCF-7, MDA-MB-231, MDA-MB-436, MDA-MB-453, MDA-MB-468) and HEK293, we confirmed uniform expression across cell lines (Fig. 1A). A panel of chemical compounds that covered various essential mechanisms was used to screen for inhibitors that could reduce RAD51 expression in wt*BRCA1/2* TNBC MDA-MB-231 and MDA-MB-453 cell lines, which was confirmed by TSO500 NGS analysis (Table S4). The NGS analysis identified the presence of sequence variation in the *NBN* and *PIK3CA* genes for MDA-MB-231, and in the *PTEN* and *TP53* genes for MDA-MB-453 (Table S4). The results from the screen identified 8 chemicals that appeared to reduce the EGFP signal (Fig. 1B). Determining the effect of these chemicals on cell viability, we found to our surprise 7 of these inhibitors actually induced acute toxicity (Fig. 1C), resulting in reduced RAD51-EGFP signal. Ivabradine (IVA) was the only inhibitor that did not affect cell viability to limit the EGFP signal, and was thus the only drug we found that most likely modulates RAD51 expression. IVA treatment led to significant reduction of RAD51-EGFP signals (Fig. 1D) in all 5 breast cancer cell lines that overexpressed HCN2 or HCN3, but not in HEK293. Western blot employed to determine the expression of endogenous HCN2, HCN3, BRCA1, BRCA2, and RAD51, confirmed that either HCN2 or HCN3 was overexpressed in the breast cancer cells (Fig. 1E, F), whilst HEK293 had low expression in both of HCN2 and HCN3 (Fig. 1E, F). The expression of BRCA1 in breast cancer cell lines, with the exception of MDA-MB-436 for which it was undetectable, was lower than that in HEK293 (Fig. 1E, F). BRCA2 expression in MCF-7, MDA-MB-231 and MDA-MB-468 was also lower than that in HEK293 while that in MDA-MB-453 and MDA-MB-468 were similar to HEK293 (Fig. 1E, F). For RAD51, the expression in all the breast cancer cell lines were similar to HEK293. The decreased RAD51 expression in MDA-MB-468 was not significant (Fig. 1E, F. By continuously monitoring the expression of EGFP-tagged RAD51 in live MDA-MB-231 and MDA-MB-453 cells, we showed that IVA treatment destabilized RAD51-EGFP (Fig. 1G). These findings suggest that IVA-mediates the down-regulation of RAD51. We hypothesized this might impact HR activity and induce BRCAness and thus sensitize breast cancer cells to PARP inhibitors.

### IVA synergized with PARP inhibitor OLA to induce synthetic lethality in TNBC

**Fig. 2:**
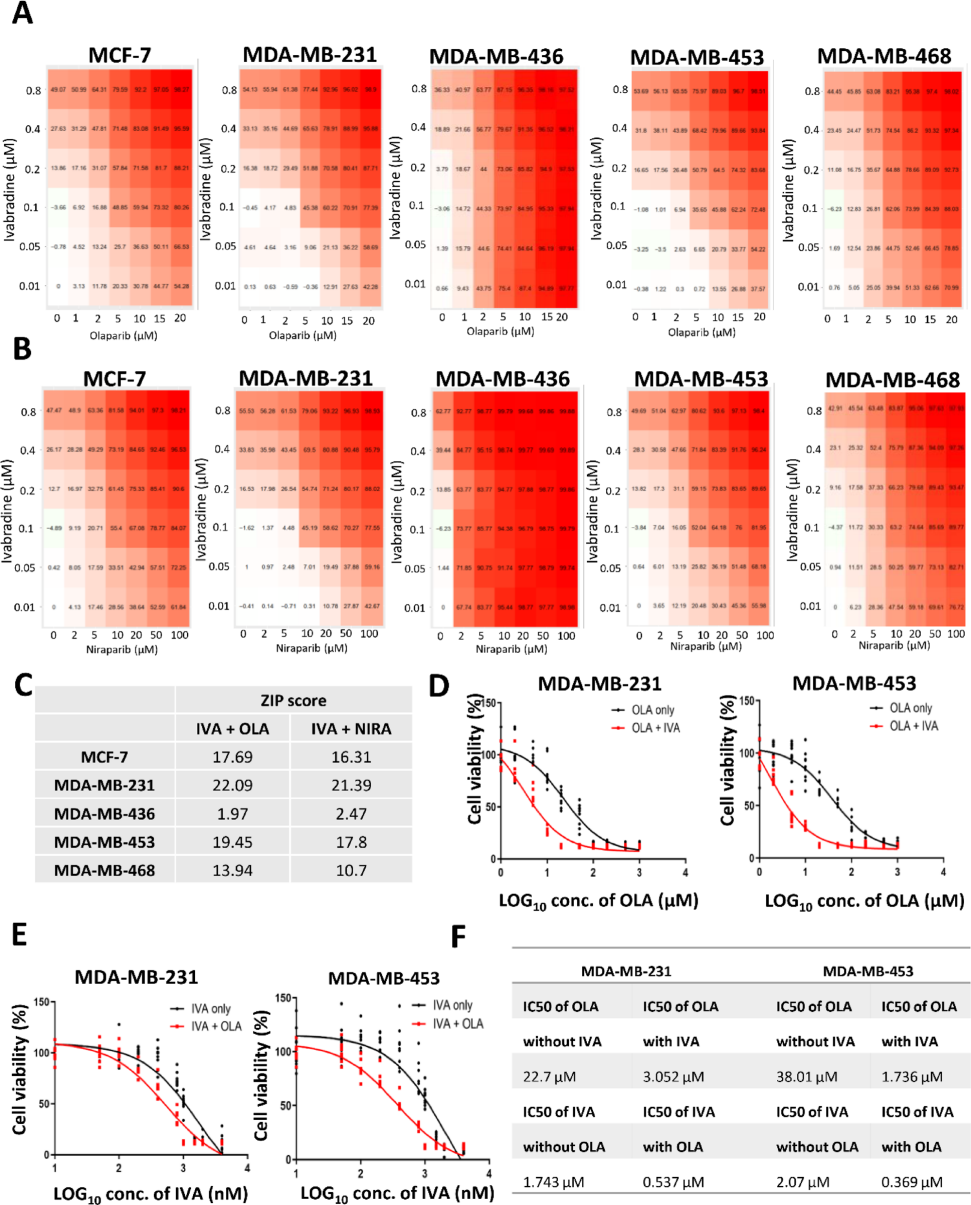
The synergistic effect of IVA and OLA. **A** the effect of different concentrations of IVA and OLA on cell viability. 5 breast cancer cell lines were examined. The cells were treated for 72 h. CCK8 was used for cell viability assay. The mean percentage of the inhibition from 5 dependent experiments was shown. **B** the effect of different concentrations of IVA and niraparib (NIRA) on cell viability. **C** ZIP scores in the 5 breast cancer cells. ZIP score was calculated using SynergyFinder 3.0. **D** Comparing the response to OLA in the presence or absence of IVA. The cells were treated with different concentrations of OLA with 0.1 µM of IVA. Cell viability was determined using CCK8 after 72 h of the treatment. Results were shown as mean ± SD from 9 independent experiments. **E** Comparing the response to IVA in the presence or absence of IVA. The cells were treated with different concentrations of IVA with 5 µM of OLA. Cell viability was determined using CCK8 after 72 h of the treatment. Results were shown as mean ± SD from 9 independent experiments. **F** The IC50 values of OLA in the presence or absence of IVA and IVA in the presence or absence of OLA were determined

We assessed the combinatorial effect of IVA with PARPi olaparib (OLA) on cell viability across 5 breast cancer cell lines. The percentage of inhibition was determined. 0.1 µM of IVA and 5 µM of OLA could inhibit cell viability by 45.36% and 35.65% in MDA-MB-231 and MDA-MB-453 respectively (Fig. 2A), which we believe these should be the minimal concentrations to achieve synergistic effect. The effect of the inhibition with the combination of IVA and another PARPi niraparib (NIRA) was also examined and similar results were obtained (Fig. 2B). The ZIP scores were determined for both combination treatments. IVA combined with OLA, showed the highest ZIP scores for wt*BRCA* TNBC cells MDA-MB-231 (22.09) and MDA-MB-453 (19.45), followed by that for wt*BRCA* ER positive cells MCF-7 (17.69) (Fig. 2C). No synergistic effect was seen for homozygous *BRCA1* deletion TNBC cell line MDA-MB-436 (ZIP score < 10 for both combination treatments). The synergistic effect for heterozygous *BRCA2* mutation TNBC cell line MDA-MB-468 was borderline (ZIP score > 10 but less than 14) (Fig. 2C).

Next, we determined the effect of OLA on cell viability in the presence of 0.1 µM of IVA in MBA-MB-231 and MDA-MB-453 cells. A left shift of the curves (red curves) was observed (Fig. 2D). Similarly, the effect of IVA on cell viability in the presence of 5 µM of OLA also showed a left shift of the curves (red curves) (Fig. 2E). By determining the IC50 values, the presence of IVA made the IC50 of OLA decrease from 22.7 to 3.052 µM and 38.01 to 1.736 µM in MDA-MB-231 and MDA-MB-453, respectively (Fig. 2F). The presence of OLA made the IC50 of IVA decrease from 1.743 to 0.537 µM and 2.07 to 0.369 µM in MDA-MB-231 and MDA-MB-453, respectively (Fig. 2F). These results confirm the synergism between IVA and OLA.

### IVA triggered RAD51 down-regulation through ER induction to compromise HR

**Fig. 3:**
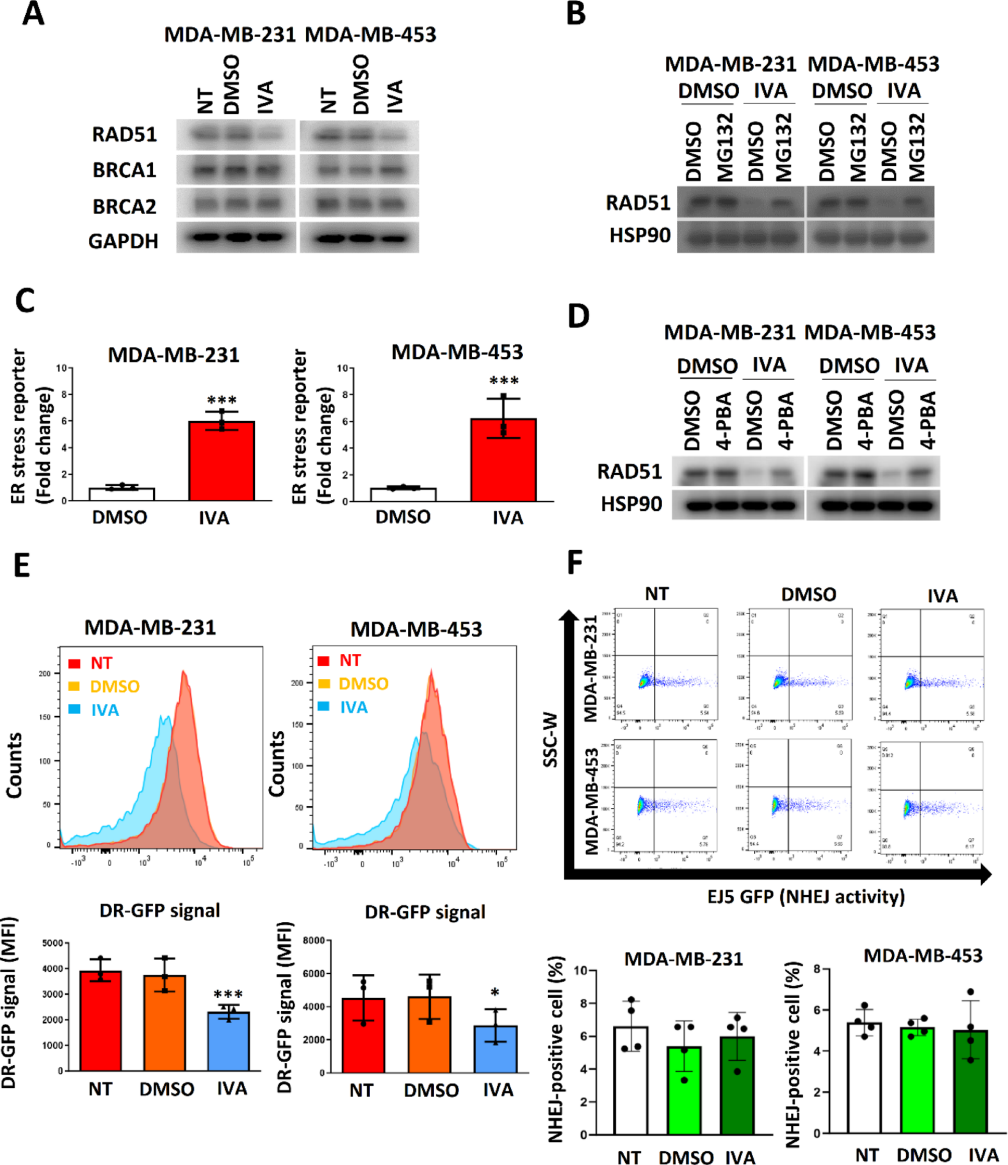
IVA compromised DNA repair mediated by HR. **A** IVA treatment reduced RAD51 expression. The cells were treated with 0.1 µM of IVA for 72 h. Western blot was employed. GAPDH was the loading control. **B** Proteasome inhibitor MG132 abolished the effect of IVA on RAD51 expression. The cells were treated with 100 nM of IVA and 5 μM of MG132 for 72 h. Western blot was performed. **C** IVA induced ER stress. The cells were treated with 0.1 µM of IVA. Luciferase reporter assay with ATF4 response element was performed 48 h post-treatment. Untreated control was used as the reference. Results were shown as mean ± SD from 3 independent experiments. **D** 4-PBA abolished the effect of IVA on RAD51 expression. The cells were treated with 0.1 µM of IVA and 10 µM of 4-PBA for 72 h. Western blot was performed. HSP90 was the loading control. **E** IVA suppressed HR. DR-GFP assay was performed. The cells were treated with 0.1 µM of IVA for 72 h. Mean Fluorescence Intensity (MFI) was determined. Results were shown as mean ± SD from 3 independent experiments. **F** IVA did not affect NHEJ. EJ5-GFP assay was performed. MDA-MB-231 and MDA-MB-453 were treated with 0.1 µM of IVA for 72 h. The GFP signal was analyzed using flow cytometry. Representative traces were shown. Results were shown as mean ± SD from 4 independent experiments. One-way ANOVA was used. NT represents no treatment control. *** represents *P* < 0.001

IVA at 0.1 µM selectively decreased RAD51 protein levels without altering *BRCA1*, *BRCA2*, or *RAD51* mRNA, indicating post-transcriptional regulation (Fig. 3A; Figure S1A,B). The decrease of RAD51 mediated by IVA was reversed by proteasome inhibitor MG132 (Fig. 3B). Therefore, IVA should enhance the protein degradation of RAD51. Luciferase reporter assay confirmed that IVA induced ER stress in MDA-MB-231 and MDA-MB-453 cells (Fig. 3C) and that the addition of ER stress reliever 4-PBA alleviated the effect of IVA on RAD51 reduction (Fig. 3D). In order to determine whether a general ER stress inducer would induce RAD51 degradation, tunicamycin (TUN) was used to treat the cells. Indeed we found TUN could induce ER stress and reduce RAD51 expression in all cell lines, including the non-cancerous HEK293 (Figure S2A). In contrast, IVA only induced ER stress and reduced RAD51 in breast cancer cell lines but had no effect on HEK293 (Figure S2B).This results highlighted the specificity of IVA. IVA compromised the efficiency of HR as indicated by pDRGFP assay using flow cytometry (Fig. 3E) while it did not affect NHEJ activity (Fig. 3F). The results from the comet assay showed that 0.1 µM of IVA did not induce double-stranded DNA breakage (DSB) (Figure S3). Together, these results suggest that 0.1 µM of IVA can specifically attenuate HR by ER stress but does not appear to exceed the capacity of the repair mechanism on the genome.

### Combined IVA and OLA treatment induced significant DNA damage to reduce cell viability

**Fig. 4:**
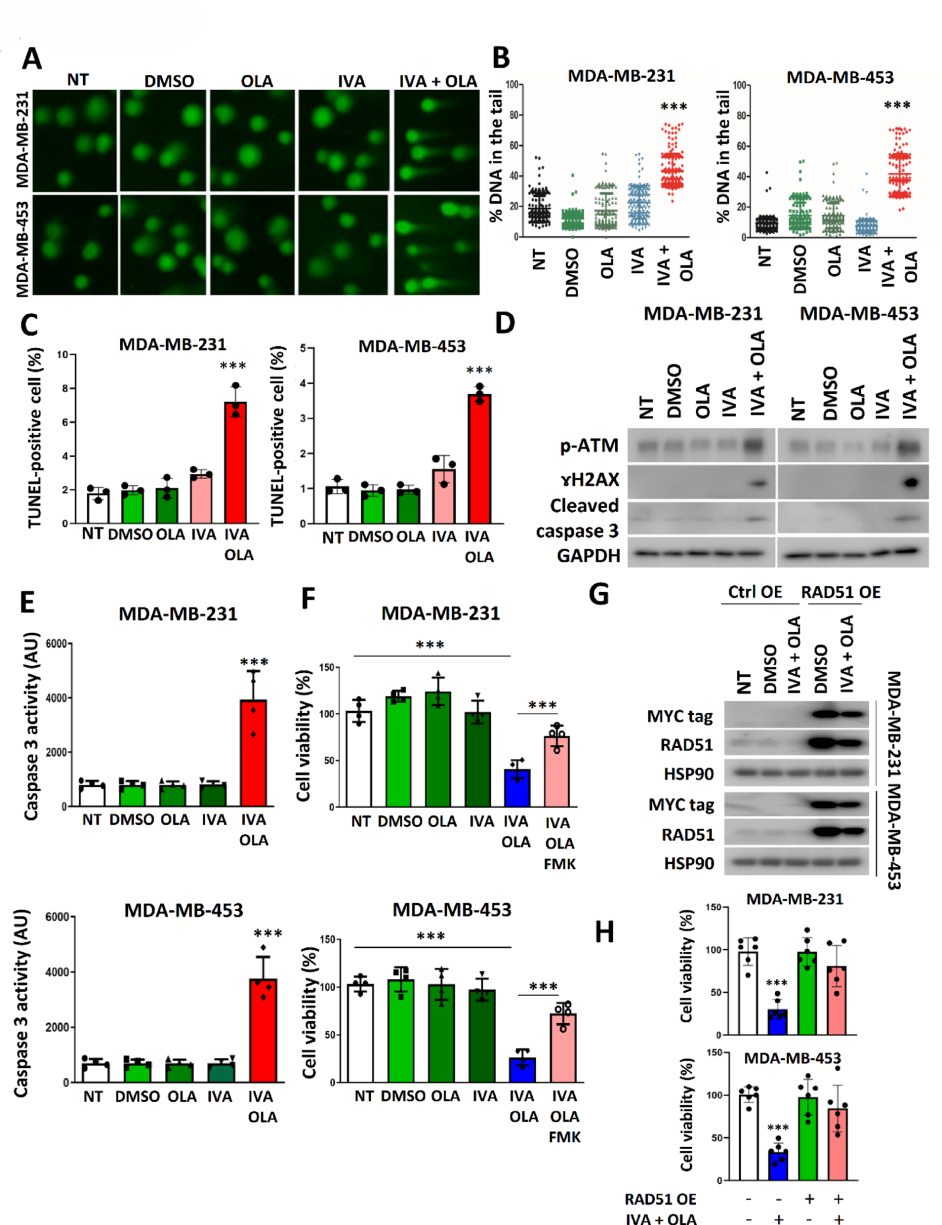
IVA and OLA co-treatment triggered DNA damage and apoptosis. **A** The co-treatment enhanced DNA damage. Comet assay was performed. **B** Statistical analysis of the comet assay in (**A**). The cells were treated with 0.1 µM of IVA and/or 5 μM of OLA for 72 h. 200 nuclei were analysed. Results were shown as mean ± SD. **C** IVA and OLA co-treatment enhanced the proportion of cells with DNA damage. TUNEL assay was performed after 72 h of treatment. Results were shown as mean ± SD from 3 independent experiments. **D** The co-treatment enhanced the expression of p-ATM and ɤH2AX, and cleaved caspase 3. Western blot was performed. **E** The co-treatment significantly enhanced the enzymatic activity of the caspase 3. Results were shown as mean ± SD from 4 independent experiments. **F** Pan-caspase inhibitor Z-VAD-FMK compromised the effect of the co-treatment on cell viability. 10 μM of Z-VAD-FMK, 0.1 µM of IVA and/or 5 μM of OLA were used for 72 h of treatment. Results were shown as mean ± SD from 4 independent experiments. **G** The effect of the co-treatment on cells expressing exogenous RAD51. The cells were transfected with *pcDNA3.1* or *pcDNA3.1_myc_RAD51*. Ctrl OE indicated control overexpression, while RAD51 OE indicated RAD51 overexpression. 24-h post-transfection, the cells were treated with 100 nM of IVA and 5 μM of OLA for 48 h. Western blot was performed to detect exogenous MYC-tagged RAD51 and total RAD51 (exogenous and endogenous). **H** RAD51 overexpression compromised the effect of the co-treatment on cell viability. The cells were transfected with *pcDNA3.1_myc_RAD51*. 24 h post-transfection, the cells were treated with 100 nM of IVA and 5 μM of OLA for 72 h. Results were shown as mean ± SD from 6 independent experiments. NT represents no treatment control. *** represent *P* < 0.001

Co-treatment with IVA and OLA led to substantial DNA damage, as shown by increased comet tails (Fig. 4A, B) and TUNEL-positive cells (Fig. 4C; Figure S4). This was accompanied by an increase in DNA damage markers (p-ATM, γH2AX) and apoptosis markers (cleaved caspase-3) (Fig. 4D). The functional assay confirmed that caspase 3 was active (Fig. 4E). The co-treatment significantly reduced cell viability, while the addition of pan caspases inhibitor Z-VAD-FMK compromised the effect of the co-treatment (Fig. 4F). Similar experiments performed on non-neoplastic cell lines MCF-10A and HEK293 showed that co-treatment did not noticeably affect RAD51, BRCA1, or BRCA2 protein levels, nor did it induce DNA damage, activated caspase 3 (Figure S5A), or cell viability (Figure S5B). Since normal breast cells have low HCN2/3 expression [8], these results indicate that the effect of both IVA and OLA are cancer cell-specific, and co-treatment has a more pronounced impact on cells that highly express HCN2/3.

Since RAD51 down-regulation may underlie the synergism between IVA and OLA, RAD51 overexpression should diminish the synergistic effect. IVA and OLA co-treatment reduced the expression of exogenous RAD51; however, even though the level of exogenous RAD51 was decreased significantly, its level was still higher than that of the control (Fig. 4G). As expected, results from cell viability confirmed that forced expression of RAD51 alleviated the effect of the co-treatment in both MDA-MB-231 and MDA-MB-453 cells (Fig. 4H). These results confirm the importance of RAD51 in the synergism mediated by the co-treatment.

### FBXO24 was essential for reducing RAD51 expression in IVA-treated breast cancer cells

**Fig. 5:**
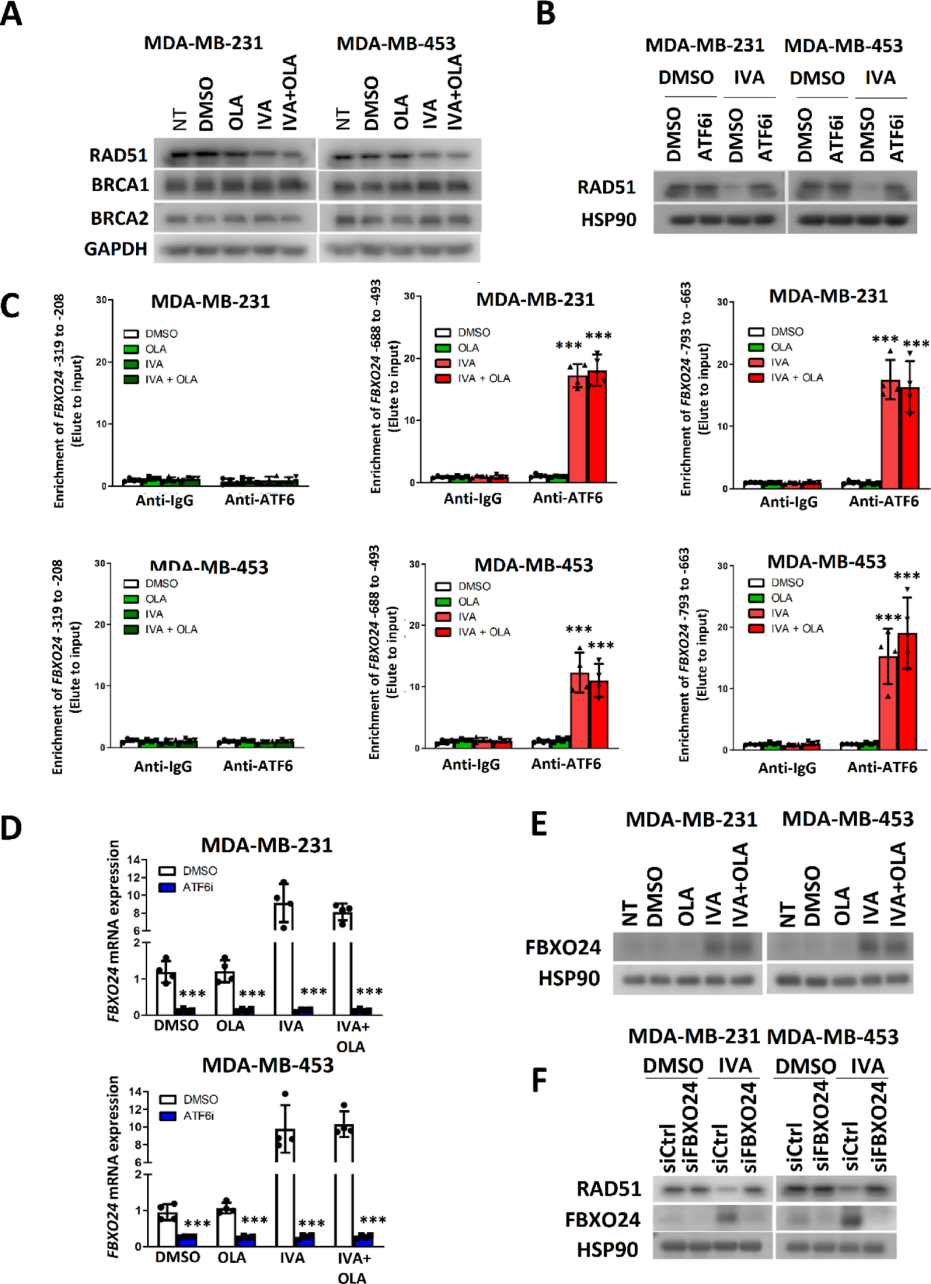
IVA employed ATF6 to induce the expression of FBXO24 to mediate RAD51 down-regulation. **A** IVA treatment and co-treatment of IVA and OLA reduced the expression of RAD51 only. The cells were treated with 0.1 µM of IVA and/or 5 μM of OLA for 72 h. Western blot was performed. GAPDH was the loading control. **B** ATF6 inhibition abolished the effect of RAD51 down-regulation mediated by IVA. 5 μM of ATF6 inhibitor Ceapin-A7, 0.1 µM of IVA and 5 μM of OLA were used. The cells were treated for 72 h. Western blot was performed. HSP90 was the loading control. **C** IVA enhanced ATF6 binding to the promoter region of *FBXO24*. The cells were treated with 0.1 µM of IVA and/or 5 μM of OLA for 72 h. ChIP was performed with anti-ATF6. qPCR was employed to determine the enrichment of 3 different regions of *FBXO24* promoter in the elute. Results were shown as mean ± SD from 4 independent experiments. One-way ANOVA was employed. **D** ATF6 inhibition abolished the effect of IVA on FBXO24 induction. 5 μM of ATF6 inhibitor Ceapin-A7 and 0.1 µM of IVA and/or 5 μM of OLA were used for treating the cells for 48 h. qPCR was performed. Results were shown as mean ± SD from 4 independent experiments. **E** IVA treatment enhanced FBXO24 protein expression. The cells were treated with 0.1 µM of IVA and/or 5 μM of OLA for 72 h. Western blot was used. HSP90 was the loading control. **F** Knockdown of *FBXO24* compromised the effect of IVA on RAD51 down-regulation. The cells were treated with 0.1 µM of IVA and 15 μM of siFBXO24 for 72 h. Western blot was used. HSP90 was the loading control. NT represents no treatment control. *** represent *P* < 0.001

Further exploration revealed that the effect of IVA on RAD51 was mediated by ATF6 activation, leading to FBXO24 upregulation. We observed that the reduction of RAD51 in cells treated with IVA was not further enhanced with the addition of OLA (Fig. 5A). Moreover, IVA and/or OLA treatment did not affect the expression level of BRCA1 and BRCA2 (Fig. 5A). Since 4-PBA compromised the effect of IVA on RAD51 (Fig. 3D), we investigated which ER stress mediator might be involved. The possible ER stress mediators PERK1, IRE1α, and ATF6 [16] were examined by inhibitor studies. We confirmed that only ATF6 inhibition reversed the effect of IVA on RAD51 expression (Fig. 5B; Figure S6A,B). Given that ATF6 is a transcription factor [17], we hypothesized that IVA might induce ATF6 to enhance the expression of an F-box protein, thus driving RAD51 protein degradation. The F-box protein provides specificity to link a substrate to E3 ubiquitin ligase to trigger protein degradation via the proteasome system [18]. By searching the whole list of ATF6 target genes (M29894) in the Molecular Signatures Database (MSigDB) [19], four F-box proteins genes were identified, ie. *FBH1*, *FBXO24*, *FBXW7*, and *FBXW11* (Table S2). By qPCR, we confirmed that IVA enhanced the *FBXO24* expression in both cell lines (Figure S7). ChIP assay also revealed that IVA treatment could enrich ATF6 at the promoter region of FBXO24 at − 688 to − 493 and at − 793 to − 633 from its transcription starting site (TSS) (Fig. 5C). qPCR confirmed that ATF6 inhibition could reduce the mRNA expression of *FBXO24*, particularly that of IVA-treated cells (Fig. 5D). As expected, the western blot confirmed that IVA treatment could enhance FBXO24 protein expression (Fig. 5E). Finally, we confirmed that *FBXO24* knockdown compromised the effect of IVA on RAD51 expression (Fig. 5F; Figure S8). In addition, we found that 4-PBA alleviated the activity of ATF6 on FBXO24 induction (Figure S9A-C). These results confirm that IVA induced ER stress through activation of ATF6 which modulated FBXO24 expression. The results also highlight that FBXO24 appears essential for mediating RAD51 reduction in IVA-treated cells.

**Fig. 6:**
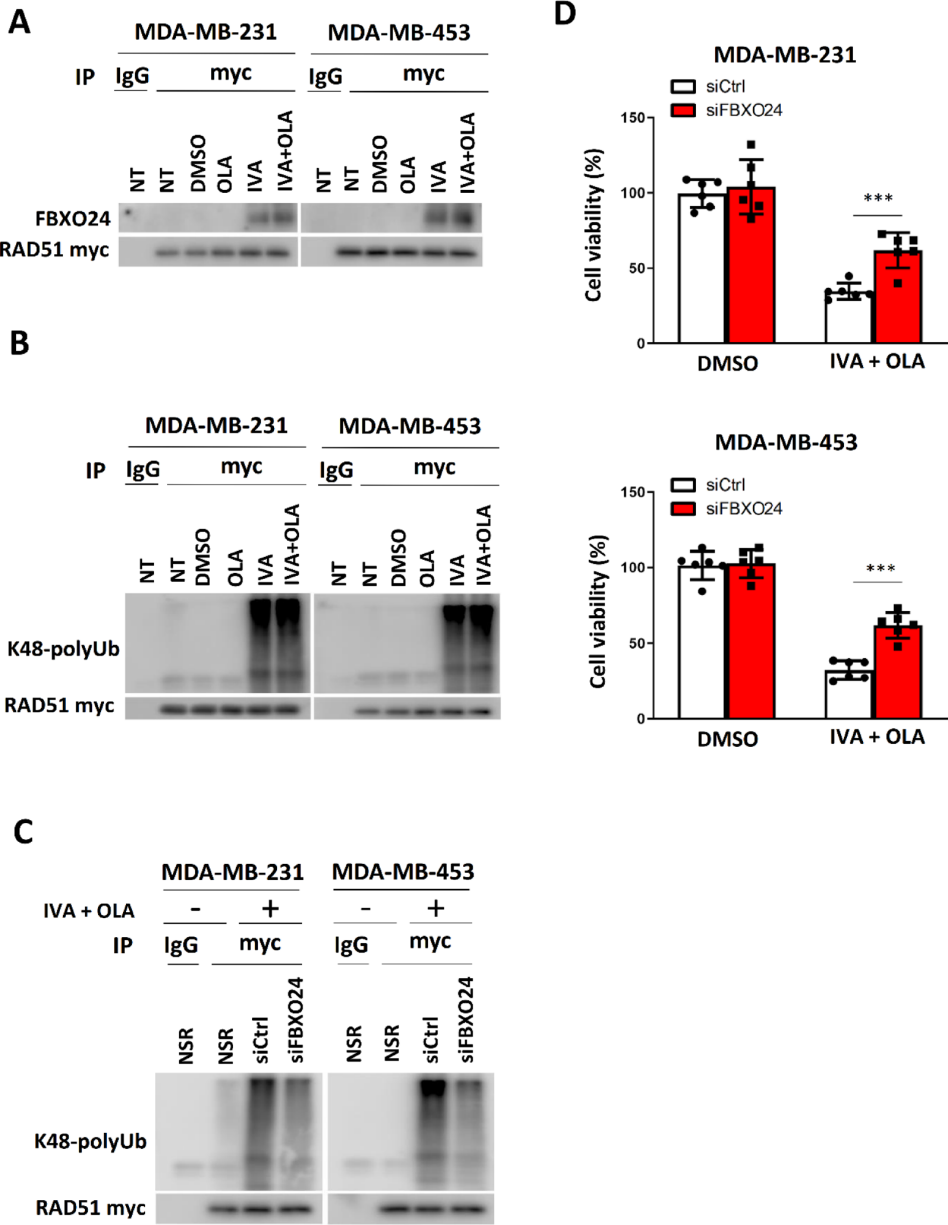
The ATF6-FBXO24 axis was essential for the efficacy of IVA and OLA co-treatment. **A** RAD51 interacted with FBXO24. The cells were transfected with *pcDNA3.1_myc_RAD51*. 24 h post-transfection, the cells were treated with 0.1 µM of IVA and/or 5 μM of OLA for 48 h. CoIP was performed with either anti-mouse IgG or anti-myc antibodies. Western blot was employed to evaluate the level of indicated protein candidates in the immunoprecipitant. **B** IVA treatment enhanced the degree of ubiquitination on RAD51. The cells were transfected with *pcDNA3.1_myc_RAD51*. 24 h post-transfection, the cells were treated with 5 μM of MG132, 0.1 µM of IVA and/or 5 μM of OLA for 48 h. Co-immunoprecipitation was performed with either anti-mouse IgG or anti-MYC antibodies. Western blot was performed using an anti-K48-linkage polyubiquitin antibody. **C** Knockdown of FBXO24 could compromise the effect of the co-treatment on the degree of K-48 conjugation polyubiquitination of RAD51. The cells were transfected with *pcDNA3.1_myc_RAD51*. 24 h post-transfection, the cells were treated with 5 μM of MG132, 15 μM of siCtrl or siFBXO24, 0.1 µM of IVA and 5 μM of OLA for 48 h. Co-immunoprecipitation was performed with either anti-mouse IgG or anti-myc antibodies. Western blot was performed using an anti-K48-linkage polyubiquitin antibody. **D** *FBXO24* knockdown weakened the efficacy of IVA and OLA co-treatment. The cells were treated with 0.1 µM of IVA, 5 μM of OLA and 15 μM of siFBXO24 or 15 μM of siCtrl for 72 h. Cell viability was assayed with CCK8. Results were shown as mean ± SD from 6 independent experiments. Students’ t-test was used. NT represents no chemical treatment. NSR represents no siRNA treatment. *** represent *P* < 0.001

Having shown that ER stress induced by IVA treatment, leading to ATF6 activation and enhanced FBXO24 expression, we further hypothesized that IVA treatment would favor the interaction between FBXO24 and RAD51 as confirmed by co-immunoprecipitation (Fig. 6A) and that this interaction underlies RAD51 degradation. Indeed, the ubiquitination assay confirmed that IVA treatment enhanced the degree of K-48 poly-ubiquitination on myc-tagged RAD51 (Fig. 6B). Similar assay was performed on the cells with *FBXO24* knockdown. The results indicated that the knockdown could reduce the degree of K48 poly-ubiquitination on the ectopic RAD51 mediated by IVA (Fig. 6C). In addition, we performed western blot on whole cell lysates from the cells with the treatment. The results showed that the total Ub signals were similar among the different treatments (Figure S10), highlighting that IVA did not impact on the whole UPS. These findings favor the idea that FBXO24 promoted RAD51 degradation via the ubiquitin–proteasome system and that IVA-induced RAD51 degradation contributes to its synergistic effect with OLA by compromising HR. The results from cell viability assays confirmed that inhibition of ATF6 (Figure S11) and knockdown of *FBXO24* (Fig. 6D) could each diminish the synergistic effect of IVA and OLA. The effect of ATF6i (Figure S11) had a stronger effect on combating the tumor suppressive effect of IVA + OLA than siFBXO24 (Fig. 6D). ATF6i is a small molecule inhibitor while siFBXO24 is a siRNA. Since the knockdown efficiency of siFBXO24 was not 100%, the residual FBXO24 could still mediate some RAD51 degradation, leading to cell death. On the other hand, ATF6 is an upstream mediator of ATF6 and ATF6i had a potent inhibitory effect. As expected, ATF6i demonstrated a stronger effect in compromising the treatment effect of IVA + OLA (Figure S11). These results confirm that IVA triggers RAD51 degradation via the ATF6-FBXO24 axis, and RAD51 reduction contributes towards boosting the efficacy of OLA.

### IVA and OLA synergistically suppress tumor growth in vivo

**Fig. 7:**
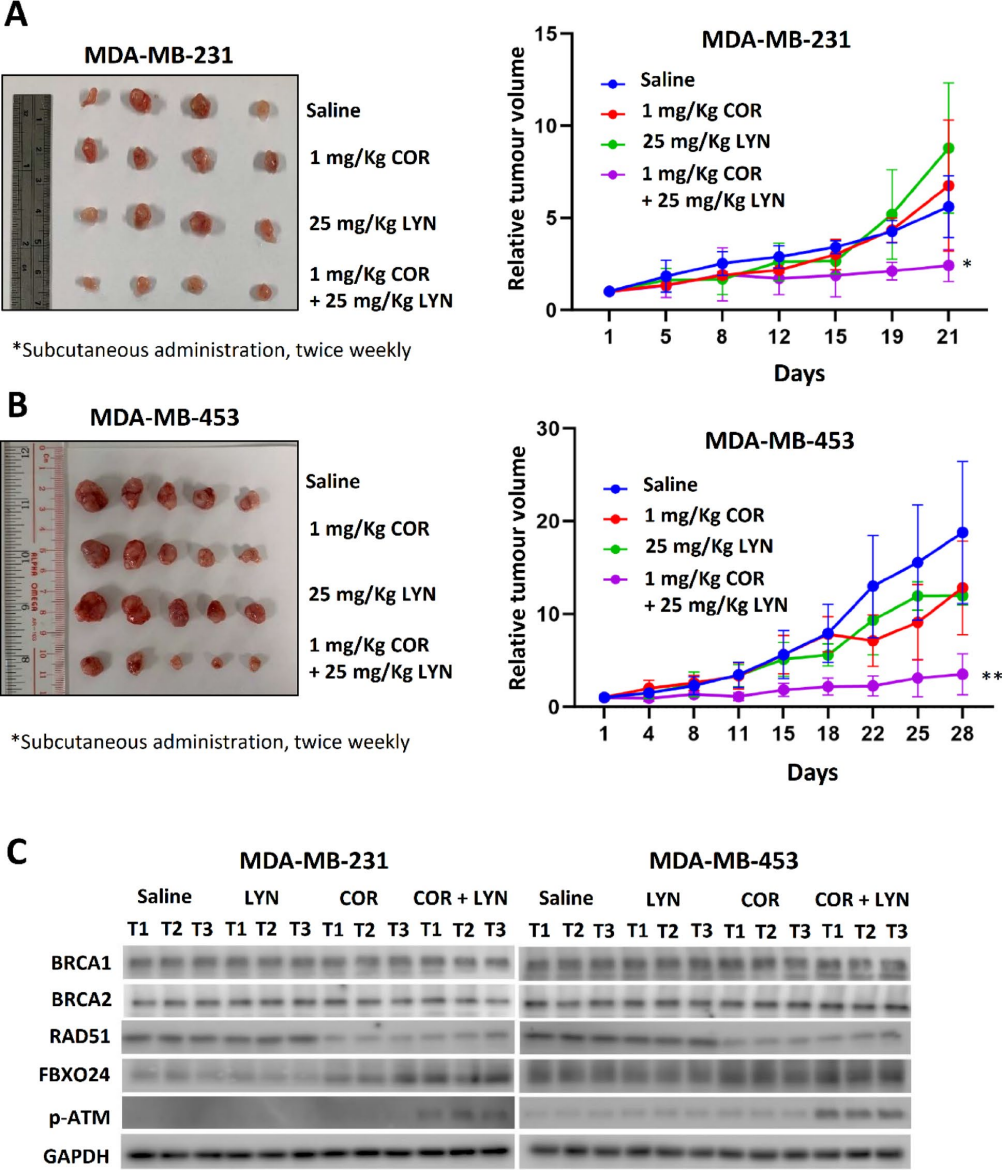
Co-treatment of COR and LYN significantly suppressed tumor growth in nude mice. The xenografts were established from **A** MDA-MB-231 and **B** MDA-MB-453. The nude mice were treated with 1 mg/Kg of COR (clinical grade Ivabradine) and 25 mg/Kg of LYN (clinical grade Olaparib) via subcutaneous injection twice a week. The volume change of the tumors was monitored. Tumor volume relative to day 1 was plotted. Results were shown as mean ± SD from 4 or 5 independent tumors. Two-way ANOVA was employed. **C** Western blot confirmed that COR treatment reduced RAD51 expression but enhanced FBXO24 expression. Phosphorylated ATM (p-ATM) level was only increased in tumors from the mice receiving the co-treatment. Protein lysates from three independent tumors from each group were analyzed. GAPDH was the loading control. * and ** represent *P* < 0.05 and *P* < 0.01, respectively

To further corroborate our in vitro findings, in vivo xenograft models were used to investigate whether IVA and OLA co-treatment would suppress tumor growth in non-g*BRCA* mutated breast cancer. Clinical grade IVA (COR) and OLA (LYN) were employed. The results showed that co-treatment of COR and LYN significantly reduced the tumor volume of xenografts established from MDA-MB-231 (Fig. 7A; Figure S12) and MDA-MB-453 (Fig. 7B; Figure S12) cell lines. Consistent with in vitro studies, COR treatment enhanced FBXO24 expression and reduced RAD51 expression (Fig. 7C). As expected, the DNA damage marker p-ATM level was only enhanced in the co-treatment group (Fig. 7C).

**Fig. 8:**
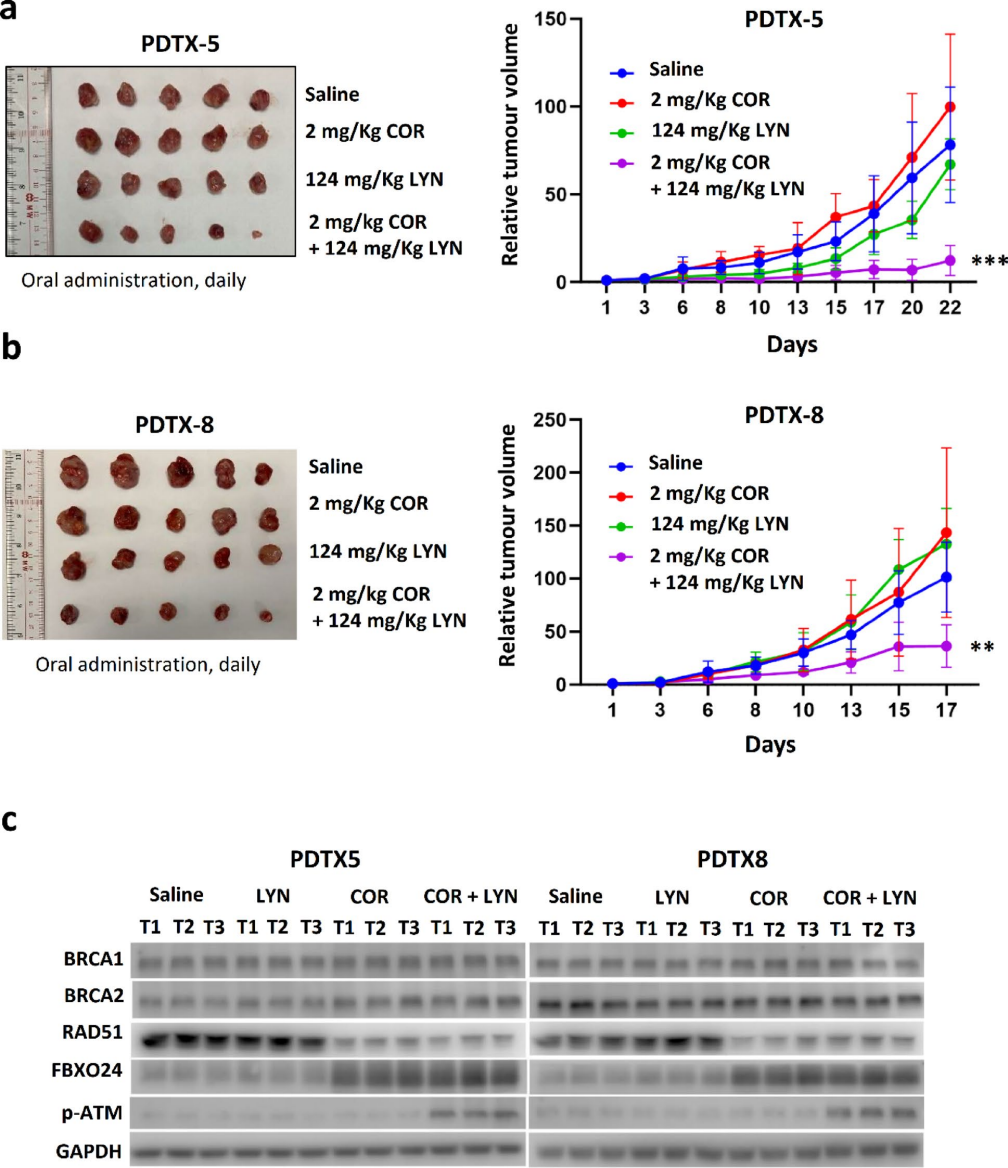
Co-treatment of COR and LYN significantly suppressed tumor growth in patient-derived tumor xenograft (PDTX) models. Two PDTX models, **A** PDTX5 and **B** PDTX8, were used. The mice were administered 2 mg/Kg of COR (clinical grade Ivabradine) and 124 mg/Kg of LYN (clinical grade Olaparib) via gavaged feeding daily. Tumor volume relative to day 1 was plotted. Results were shown as mean ± SD from 5 independent tumors. Two-way ANOVA was employed. **C** Western blot confirmed that COR treatment reduced the expression of RAD51 but enhanced FBXO24 expression. Phosphorylated ATM (p-ATM) level was only increased in tumors from the mice receiving the co-treatment. Protein lysates from three independent tumors from each group were analyzed. GAPDH was the loading control. ** and *** represent *P* < 0.01 and *P* < 0.001, respectively

In addition, the effect of COR and LYN was further tested on TNBC patient-derived xenografts (PDTXs), which were established in our previous study. Two independent PDTX models, PDTX5 and PDTX8, were selected based on the absent germline mutation status of *BRCA1/2*. These two models were previously found to be responsive to IVA, and each was confirmed to have high HCN2 and HCN3 expression [8]. Sanger sequencing of the coding sequences and exon–intron junctions of *BRCA1* and *BRCA2* on DNA from the blood samples of the patients who donated their breast cancer tissue for PDTX, had confirmed the absence of pathogenic germline mutations in both PDTX models (Tables S3 and S4). COR and LYN were given by oral administration. The results showed that the co-treatment significantly reduced tumor volume (Fig. 8A, B; Figure S13). Western blot confirmed that COR treatment resulted in down-regulation of RAD51 and up-regulation of FBXO24 in the tumors (Fig. 8C). Similar to the results from the nude mice study, only the co-treatment could enhance the level of p-ATM (Fig. 8C). Together, these findings highlight that co-treatment of COR and LYN in the non-germline *BRCA* mutated breast cancer effectively reduced tumor volume.

## Discussion

TNBC remains a challenging malignancy due to limited targeted therapies and poor prognosis [20]. While chemotherapy and emerging neoadjuvant chemoimmunotherapy can be offered, they are often associated with significant toxicity and relapse. PARPi treatement, such as OLA has shown promise in BRCA-mutated cancers by exploiting synthetic lethality in HR-deficient tumors [21]. However, their efficacy is limited in non-g*BRCA*m TNBC [22–24], and acquired resistance remains a significant hurdle [25, 26]. This underscores the need for rational drug combinations that enhance PARPi efficacy while minimizing toxicity.

This study identified IVA, an FDA-approved HCN channel blocker, as a novel agent that sensitized non-g*BRCA*m TNBC to PARPi. IVA induced RAD51 degradation (Fig. 1), a critical mediator of HR repair. This mechanism compromised HR efficiency (Fig. 3), leading to synthetic lethality when combined with PARPi (Fig. 2). Our findings demonstrates that IVA reduced RAD51 protein levels by inducing ER stress (Fig. 3A-D), thus impairing HR repair, resulting in increased DNA damage and apoptosis in *BRCA*-proficient TNBC cells (Fig. 4). Although the ER stress inducer TUN can induce ER stress and reduce RAD51, the effect of TUN is not specific for breast cancer cells only, and is thus unsuitable for use as a treatment regiment. On contrast, IVA induces ER stress in breast cancer cells with increased HCN2/HCN3 expression, which is not found in non-neoplastic cell line HEK293. Hence IVA can be used with PARP, specifically for breast cancers that express HCN2/HCN3 to induce synergistic effect. Importantly, overexpression of RAD51 abrogated the synergistic effects of IVA and OLA, confirming RAD51’s central role in this therapeutic strategy (Fig. 4G, H). The concept of BRCAness describes the functional state of HR deficiency, mimicking the effects of *BRCA1/2* mutations and conferring sensitivity to PARPi [27]. By demonstrating the ability of IVA to impair HR, we propose that IVA acts as an inducer of BRCAness in non-g*BRCA*m TNBC, thereby sensitizing these cells to PARP inhibition.

Mechanistically, IVA activated ATF6 (Fig. 5), which upregulated FBXO24 (Fig. 6), an E3 ubiquitin ligase [28–30] that targeted RAD51 for degradation. This novel ATF6-FBXO24-RAD51 axis provides the molecular basis for IVA-induced BRCAness. In vivo, the IVA-OLA combination significantly suppressed tumor growth in both cell line-derived (Fig. 7) and PDTX (Fig. 8) models. These findings highlight the translational potential of repurposing IVA to enhance PARPi efficacy in non-g*BRCA*m TNBC.

Our drug screening results further corroborated these findings, as it demonstrated that Ceapin-A7, an ATF6 inhibitor, increased RAD51-EGFP levels (Fig. 1B, 10th row). This observation provides the first evidence implicating ATF6 in regulating RAD51 expression, prompting further investigation into the underlying molecular mechanism. Given that IVA modulated RAD51 protein stability, we explored potential mediators of RAD51 degradation that might be regulated by ATF6.

The recommended maximal oral dosages for COR and LYN (clinical grade IVA and OLA) are 0.375 mg/Kg/day and 15 mg/Kg/day, respectively, in human subjects. Based on the human-mouse equivalent dose conversion [31], the equivalent maximum dosages in mice would be 4.61 mg/kg/day for COR and 184.50 mg/kg/day for LYN. In this study, we administered COR at 2 mg/kg/day and LYN at 124 mg/kg/day orally, both below the calculated maximum equivalent doses. Importantly, we observed no overt adverse effects or significant body weight loss in mice treated with either subcutaneous injection or oral administration of the drug combination, suggesting that the chosen dosages and combination were well-tolerated. While our data suggest a favorable safety profile at the administered doses, the escalation of IVA or OLA might lead to different outcomes, potentially enhancing efficacy but also increasing the risk of side effects. Thus, dose optimization studies in larger preclinical models could be essential before advancing to clinical trials. Our previous work, which included electrocardiographic monitoring of mice treated with IVA at even higher doses for four weeks, had demonstrated no significant changes in heart rate compared to controls [8], further supporting the tolerability of COR. While our current data suggests a favorable safety profile, higher dosages of COR and LYN might potentially enhance therapeutic efficacy. OLA is associated with common side effects such as anemia, neutropenia, and fatigue [32]. While other chemical inhibitors of RAD51 and HR have been reported [33–35], IVA’s favorable safety profile and FDA approval for cardiac conditions expedite its potential clinical application [36]. Despite these promising results, several considerations merit further exploration. Firstly, the long-term effects of IVA in a clinical setting, particularly concerning its cardiac effects, require thorough monitoring. Secondly, the resistance mechanisms, such as those involving reverse BRCA mutations or other compensatory pathways, need to be investigated to understand potential escape routes from this therapeutic strategy.

PARPi resistance often arises through HR restoration or replication fork stabilization [25]. Restoration of HR, for instance, through reverse *BRCA2* mutations leading to functional *BRCA2* protein recovery [37], is a well-documented resistance mechanism. *BRCA2* plays a crucial role in HR-mediated DNA repair by interacting with the RAD51 recombinase, thereby maintaining genome integrity [38]. RAD51 itself is a critical factor in DNA replication, repair, and recombination [39], particularly in the repair of DSBs via HR [15], Overexpression of RAD51 has been observed in various cancers [40], and is associated with poor outcomes in breast cancer patients [41], underscoring its clinical significance. While previous studies have shown that RAD51 inhibition can enhance the efficacy of radiotherapy [42], further investigation into the precise mechanisms and extent of DNA damage is warranted.

While *BRCA* status and the Homologous Recombination Deficiency score [43] are currently used to predict PARPi sensitivity in breast cancers, predictive biomarkers for response to PARPi combination therapies remain limited, hindering clinical translation. This study convincingly demonstrates the synergistic anti-tumor activity of the HCN channel blocker IVA in combination with the PARPi OLA. Our work also suggests that HCN2/3 expression, which is elevated in a significant proportion of TNBC tumors, may serve as a predictive biomarker for IVA-OLA combination therapy [8]. This could enable personalized treatment strategies for non-g*BRCA*m TNBC patients with high HCN expression.

## Conclusions

This study has uncovered a novel therapeutic strategy that leverages the ATF6-FBXO24-RAD51 axis to induce BRCAness and sensitize non-g*BRCA*m TNBC to PARPi. The IVA-PARPi combination offers a promising approach to expand PARPi utility and address the unmet need in this aggressive breast cancer subtype. Future research should focus on refining this combination for clinical application, exploring biomarkers for patient stratification, and addressing potential resistance mechanisms to ensure broader therapeutic benefits.

## Supplementary Information

Below is the link to the electronic supplementary material.

Supplementary Material 1

Supplementary Material 2

Supplementary Material 3

Supplementary Material 4

Supplementary Material 5

Supplementary Material 6

Supplementary Material 7

## Data Availability

The datasets used and/or analysed during the current study are available from the corresponding author on reasonable request.

## Abbreviations

COR: Coralan
DSB: DNA double-strand break
g*BRCA*m: Germline-*BRCA* mutation
HCN: Hyperpolarisation-activated cyclic nucleotide-gated channel
HR: Homologous recombination
IVA: Ivabradine
LYN: Lynparza
MSigDB: Molecular signatures database
OLA: Olaparib
PARPi: PARP inhibitors
PDTX: Patient-derived tumour xenograft
qPCR: Quantitative real time polymerase chain reaction
TSS: Transcription starting site
TNBC: Triple-negative breast cancer
