# Supplementary material for: Ivabradine induces RAD51 degradation, potentiating PARP inhibitor efficacy in non-germline BRCA pathogenic variant triple-negative breast cancer
Source: J Transl Med. 2025 Aug 5;23:860. doi: 10.1186/s12967-025-06902-8 (PMC12323259; doi:10.1186/s12967-025-06902-8)
Supplement: Supplementary file 1 — Supplementary Material 1 [file 12967_2025_6902_MOESM1_ESM.docx]

**Table S1 shows the inhibitors used**

| **Inhibitors** | **Cat. No** | **Company** |
| --- | --- | --- |
| 5-FU | HY-90006 | MedChemExpress |
| 5-HT | HY-B1473A | MedChemExpress |
| Actinomycin D | S8964 | Selleckchem |
| Anastrozole | S1188 | Selleckchem |
| BafA1 | HY-100558 | MedChemExpress |
| Bicalutamide | S1190 | Selleckchem |
| Binimetinib | S7007 | Selleckchem |
| BML-277 | S8632 | Selleckchem |
| CFI-400945 | HY-12300B | MedChemExpress |
| Clopidogrel Bisulfate | HY-17459 | MedChemExpress |
| Enzalutamide | HY-70002 | MedChemExpress |
| Fatostatin | S9785 | Selleckchem |
| Fulvastrant | HY-13636 | MedChemExpress |
| GPNA | S6670 | Selleckchem |
| GSK2606414 | S7307 | Selleckchem |
| HCQ | H0915 | Sigma |
| Ivabradine | HY-B0162A | MedChemExpress |
| JNK-IN-8 | HY-13319 | MedChemExpress |
| KI696 | E1141 | Selleckchem |
| Kira6 | S8658 | Selleckchem |
| Lamivudine | HY-B0250 | MedChemExpress |
| Lenalidomide | S1029 | Selleckchem |
| Linsitinib | S1091 | Selleckchem |
| Lomitapide Mesylate | HY-14668 | MedChemExpress |
| Lumateperone Tosylate | HY-19733 | MedChemExpress |
| MG132 | S2619 | Selleckchem |
| MK8353 | HY-111407 | MedChemExpress |
| MK8722 | HY-111363 | MedChemExpress |
| ML167 | HY-15951 | MedChemExpress |
| NR1 | HY-13756 | MedChemExpress |
| Ofloxacin | HY-B0125 | MedChemExpress |
| Olaparib | S1060 | Selleckchem |
| Panobinostat Lactate | HY-10224A | MedChemExpress |
| Rabusertib | HY-14720 | MedChemExpress |
| Rapamycin | S1039 | Selleckchem |
| Roscoritine | S1153 | Selleckchem |
| Ruxolitinib | HY-50856 | MedChemExpress |
| Saquinavir Mesylate | HY-17003 | MedChemExpress |
| Sorafinib | S1040 | Selleckchem |
| Tacrolimus | HY-13756 | MedChemExpress |
| Terbinafine Hydrochloride | HY-17395 | MedChemExpress |
| TG003 | S7320 | Selleckchem |
| Tocilizumab | HY-P9917 | MedChemExpress |
| Trazodone Hydrochloride | HY-B0478 | MedChemExpress |
| Tunicamycin | S7894 | Selleckchem |
| Vemurafenib | HY-12057 | MedChemExpress |
| Wortmaninin | HY-10197 | MedChemExpress |
