# Supplementary material for: Ivabradine induces RAD51 degradation, potentiating PARP inhibitor efficacy in non-germline BRCA pathogenic variant triple-negative breast cancer
Source: J Transl Med. 2025 Aug 5;23:860. doi: 10.1186/s12967-025-06902-8 (PMC12323259; doi:10.1186/s12967-025-06902-8)
Supplement: Supplementary file 5 — Supplementary Material 5 [file 12967_2025_6902_MOESM5_ESM.pdf]

# Figure S1

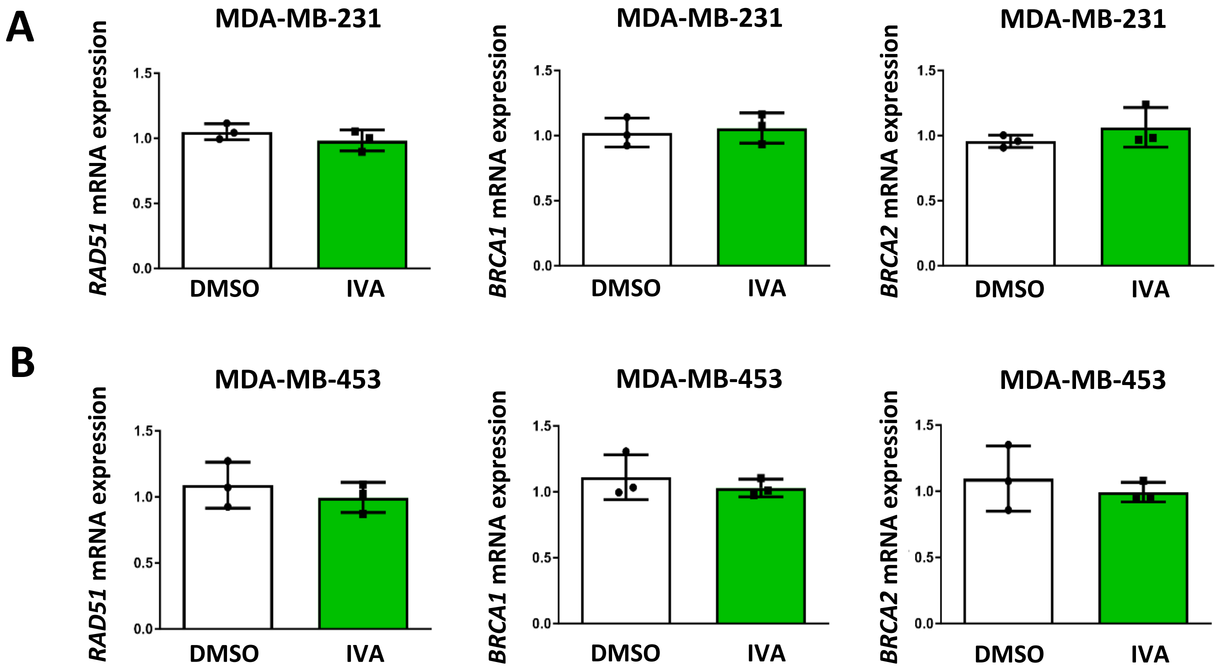
A
MDA-MB-231
RAD51 mRNA expression
1.5
1.0
0.5
0.0
DMSO
IVA
MDA-MB-231
BRCA1 mRNA expression
DMSO
IVA
MDA-MB-231
BRCA2 mRNA expression
DMSO
IVA
B
MDA-MB-453
RAD51 mRNA expression
DMSO
IVA
MDA-MB-453
BRCA1 mRNA expression
DMSO
IVA
MDA-MB-453
BRCA2 mRNA expression
DMSO
IVA

# Figure S2

**A**

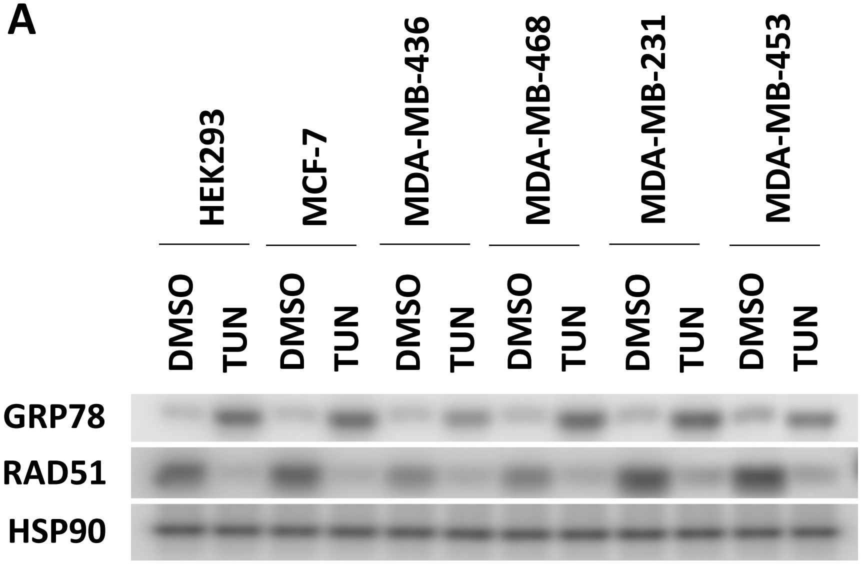
HEK293
MCF-7
MDA-MB-436
MDA-MB-468
MDA-MB-231
MDA-MB-453
DMSO
TUN
DMSO
TUN
DMSO
TUN
DMSO
TUN
DMSO
TUN
DMSO
TUN
GRP78
RAD51
HSP90

**B**

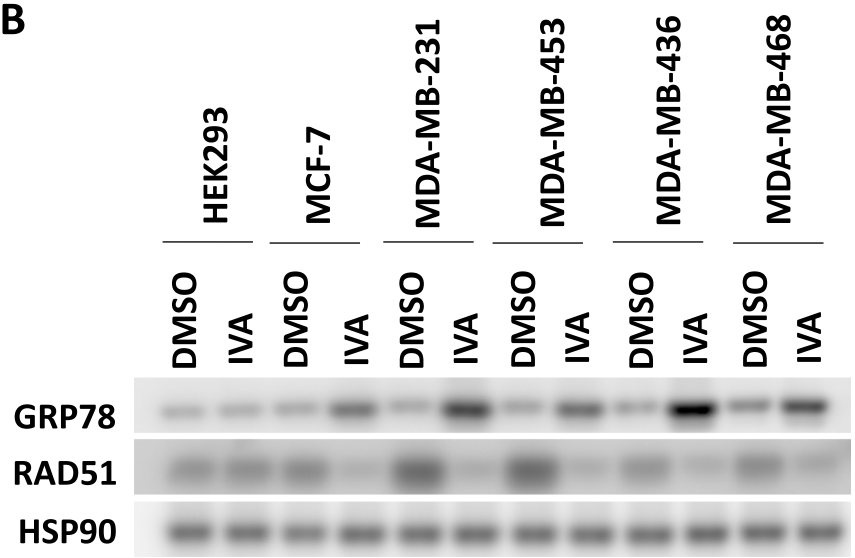
HEK293
MCF-7
MDA-MB-231
MDA-MB-453
MDA-MB-436
MDA-MB-468
DMSO
IVA
DMSO
IVA
DMSO
IVA
DMSO
IVA
DMSO
IVA
DMSO
IVA
GRP78
RAD51
HSP90

# Figure S3

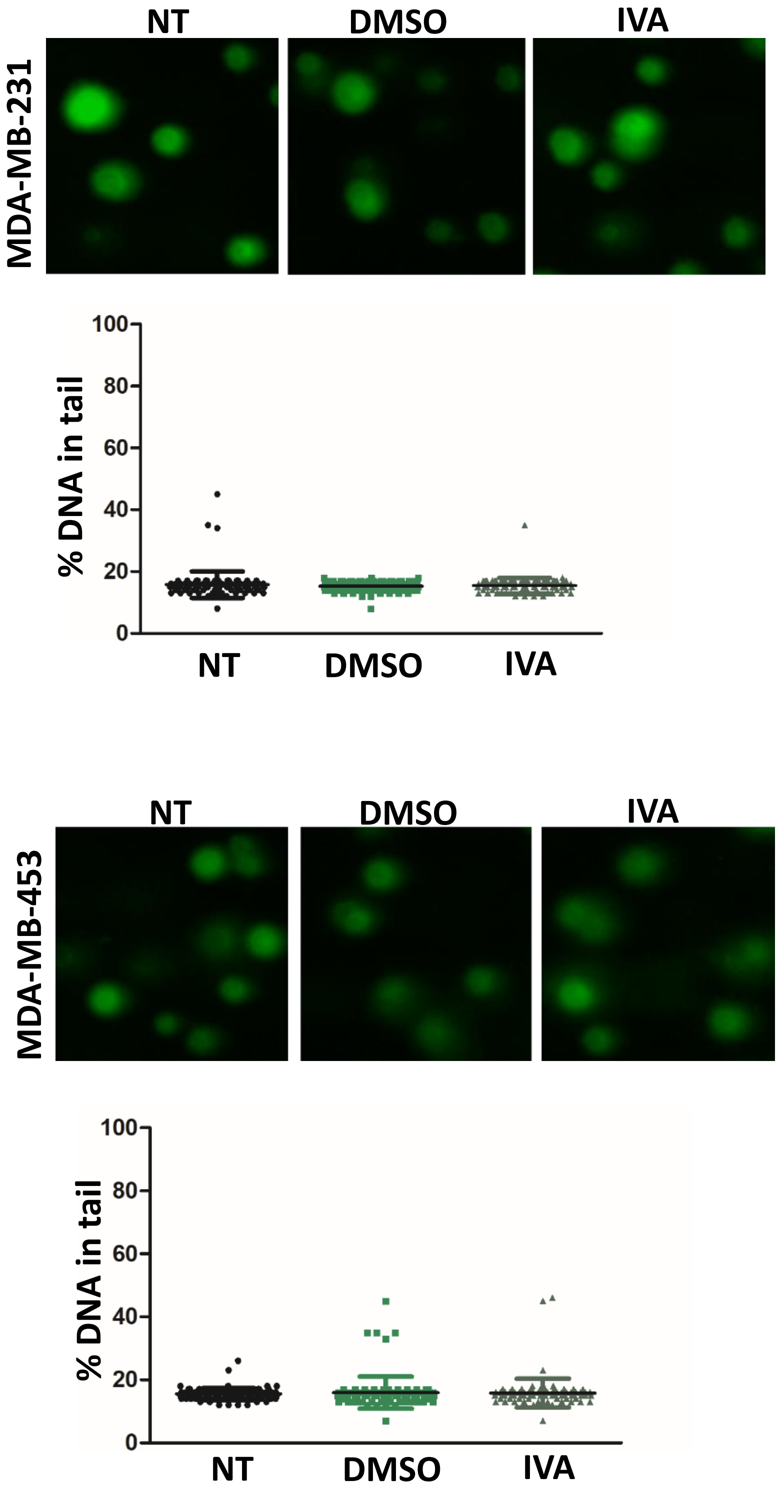
NT
DMSO
IVA
MDA-MB-231
100
80
60
40
20
0
% DNA in tail
NT
DMSO
IVA
NT
DMSO
IVA
MDA-MB-453
100
80
60
40
20
0
% DNA in tail
NT
DMSO
IVA

# Figure S4

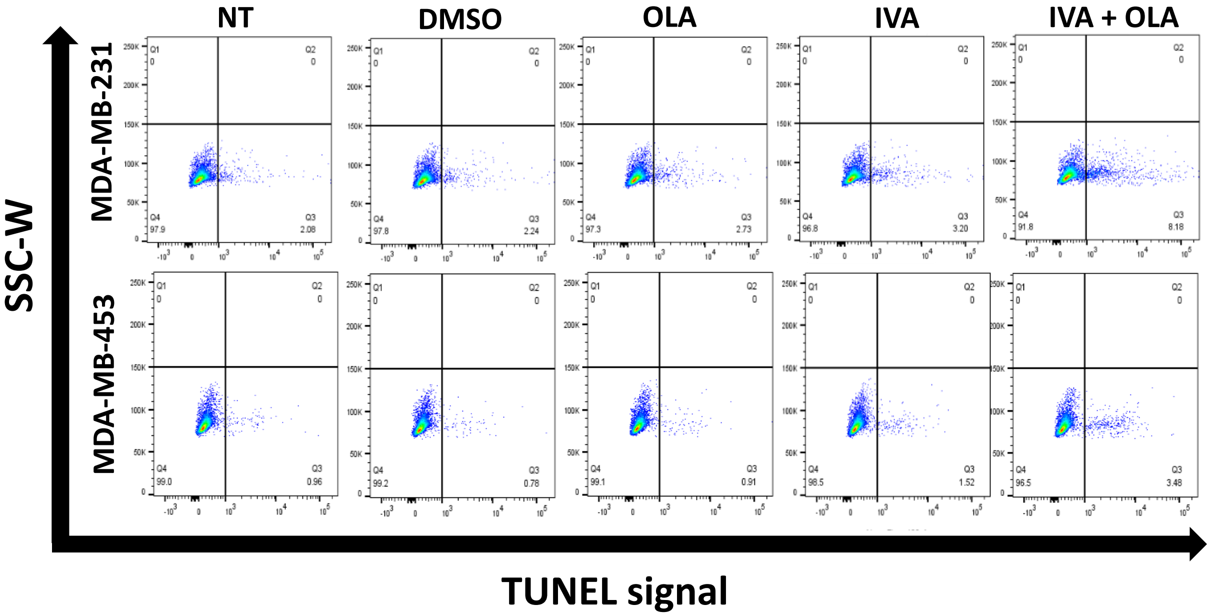

NT
DMSO
OLA
IVA
IVA + OLA
MDA-MB-231
MDA-MB-453
SSC-W
TUNEL signal
Q4 97.9 Q3 2.08
Q4 97.8 Q3 2.24
Q4 97.3 Q3 2.73
Q4 96.8 Q3 3.20
Q4 91.8 Q3 8.18
Q4 99.0 Q3 0.96
Q4 99.2 Q3 0.78
Q4 99.1 Q3 0.91
Q4 98.5 Q3 1.52
Q4 96.5 Q3 3.48

# Figure S5

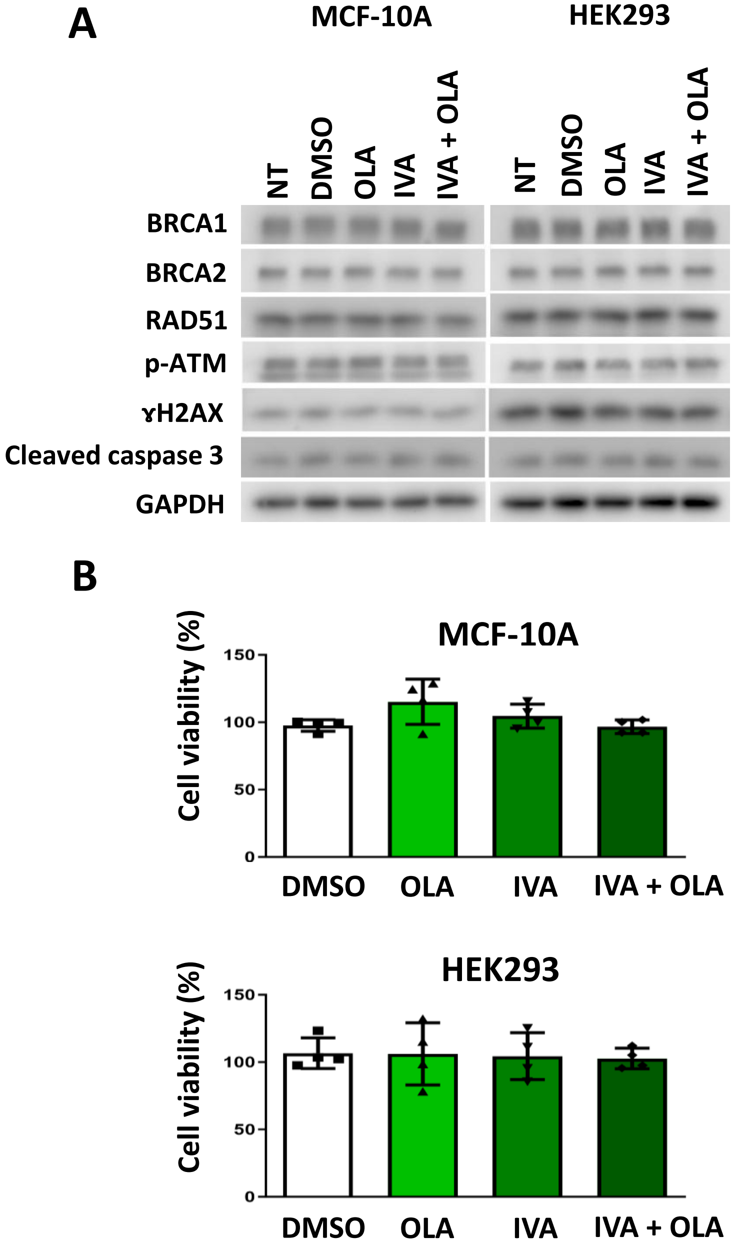
A
MCF-10A
HEK293
NT
DMSO
OLA
IVA
IVA + OLA
BRCA1
BRCA2
RAD51
p-ATM
ɣH2AX
Cleaved caspase 3
GAPDH
B
MCF-10A
Cell viability (%)
150
100
50
0
DMSO
OLA
IVA
IVA + OLA
HEK293
Cell viability (%)
150
100
50
0
DMSO
OLA
IVA
IVA + OLA

# Figure S6

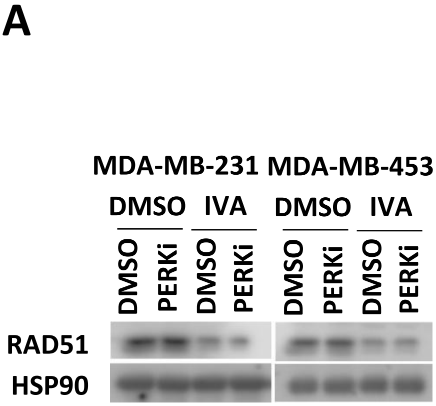
A
MDA-MB-231
MDA-MB-453
DMSO
IVA
DMSO
IVA
DMSO
PERKi
DMSO
PERKi
DMSO
PERKi
DMSO
PERKi
RAD51
HSP90

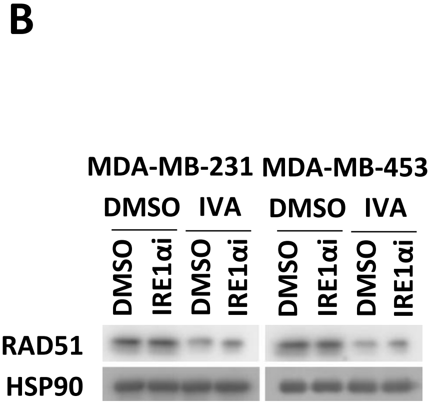
B
MDA-MB-231
MDA-MB-453
DMSO
IVA
DMSO
IVA
DMSO
IRE1αi
DMSO
IRE1αi
DMSO
IRE1αi
DMSO
IRE1αi
RAD51
HSP90

# Figure S7

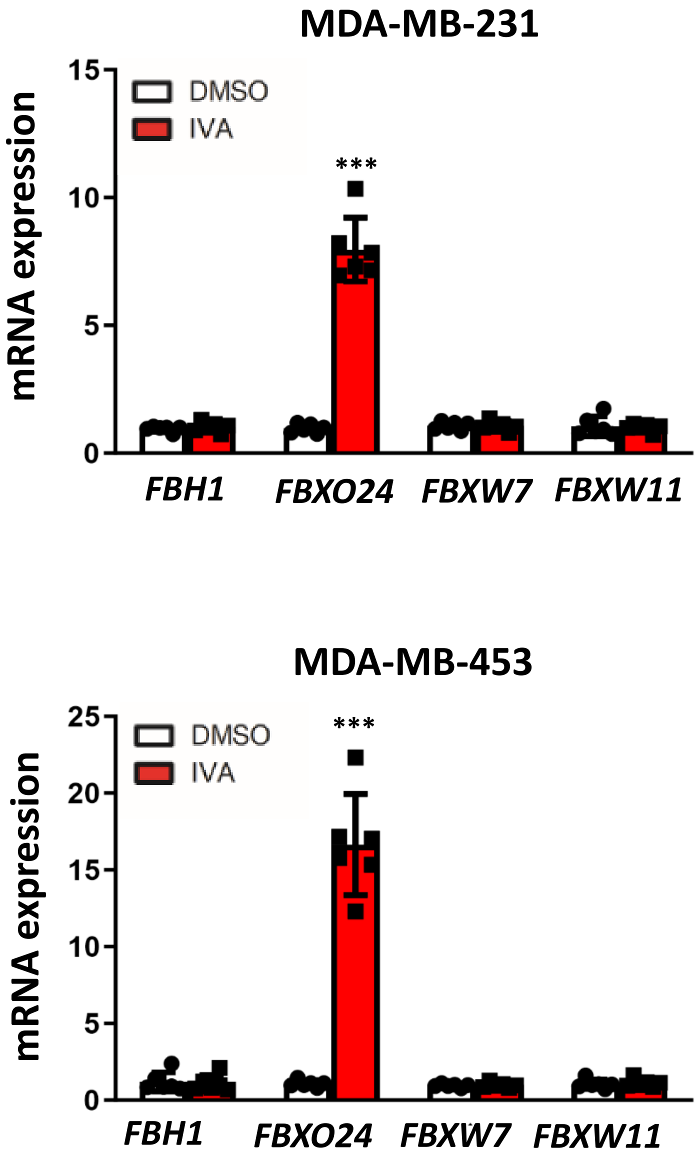
MDA-MB-231
mRNA expression
15
10
5
0
DMSO
IVA
***
FBH1
FBXO24
FBXW7
FBXW11
MDA-MB-453
mRNA expression
25
20
15
10
5
0
DMSO
IVA
***
FBH1
FBXO24
FBXW7
FBXW11

# Figure S8

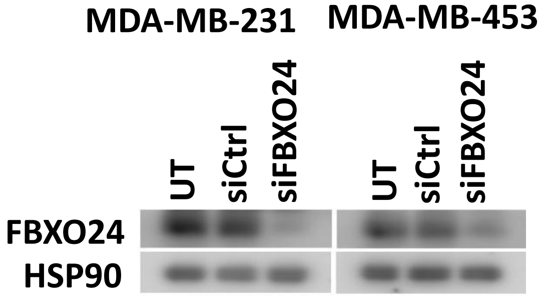
MDA-MB-231
MDA-MB-453
UT
siCtrl
siFBXO24
UT
siCtrl
siFBXO24
FBXO24
HSP90

# Figure S9

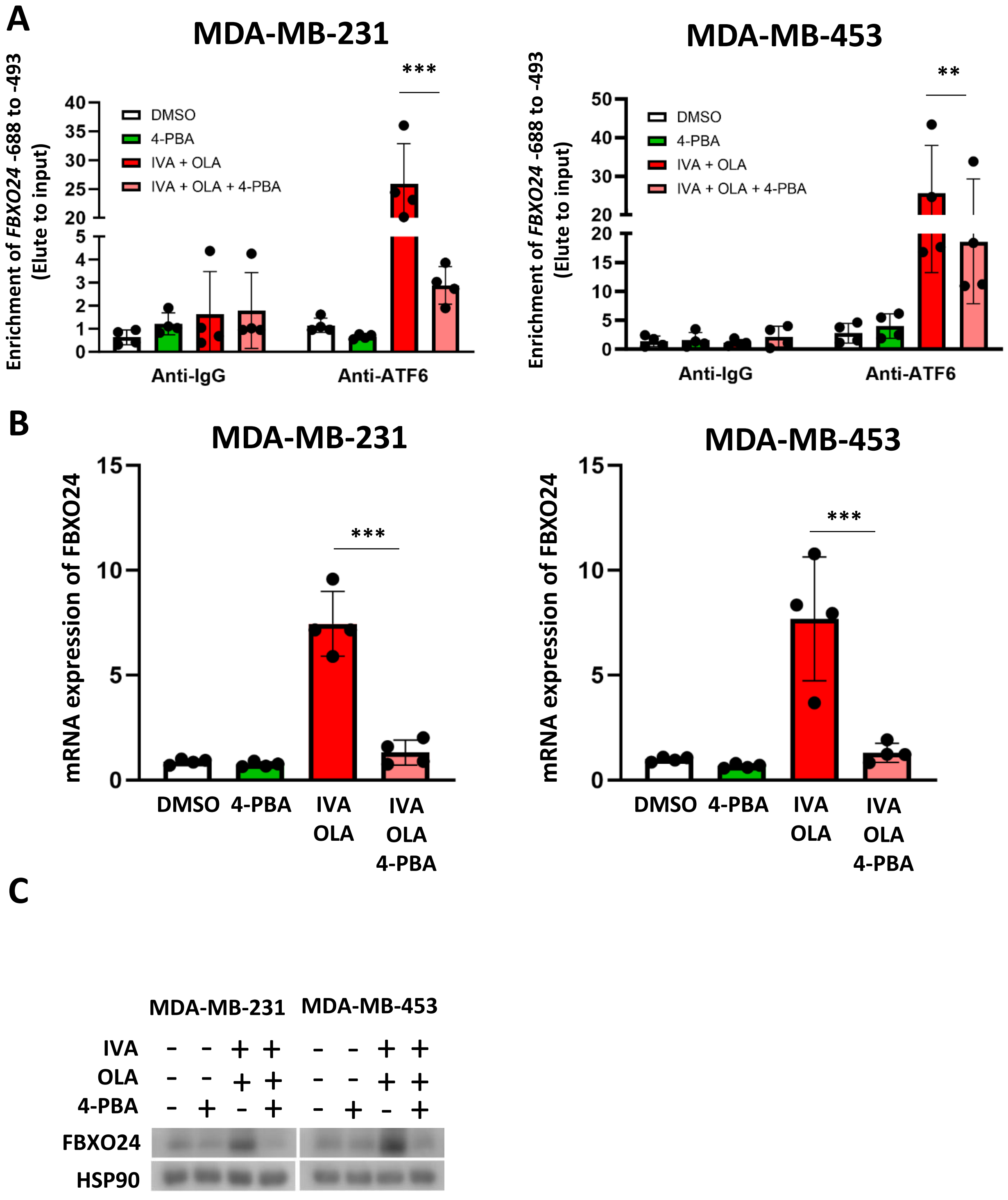

A
MDA-MB-231
MDA-MB-453
Enrichment of FBXO24 -688 to -493 (Elute to input)
DMSO
4-PBA
IVA + OLA
IVA + OLA + 4-PBA
Anti-IgG
Anti-ATF6
***
**
B
MDA-MB-231
MDA-MB-453
mRNA expression of FBXO24
DMSO
4-PBA
IVA OLA
IVA OLA 4-PBA
***
C
MDA-MB-231
MDA-MB-453
IVA
OLA
4-PBA
FBXO24
HSP90

# Figure S10

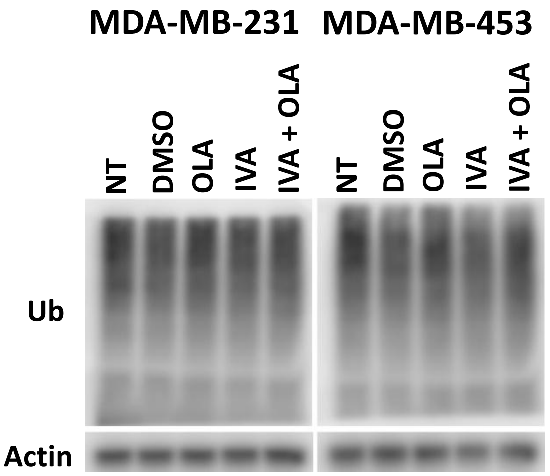
MDA-MB-231
MDA-MB-453
NT
DMSO
OLA
IVA
IVA + OLA
NT
DMSO
OLA
IVA
IVA + OLA
Ub
Actin

# Figure S11

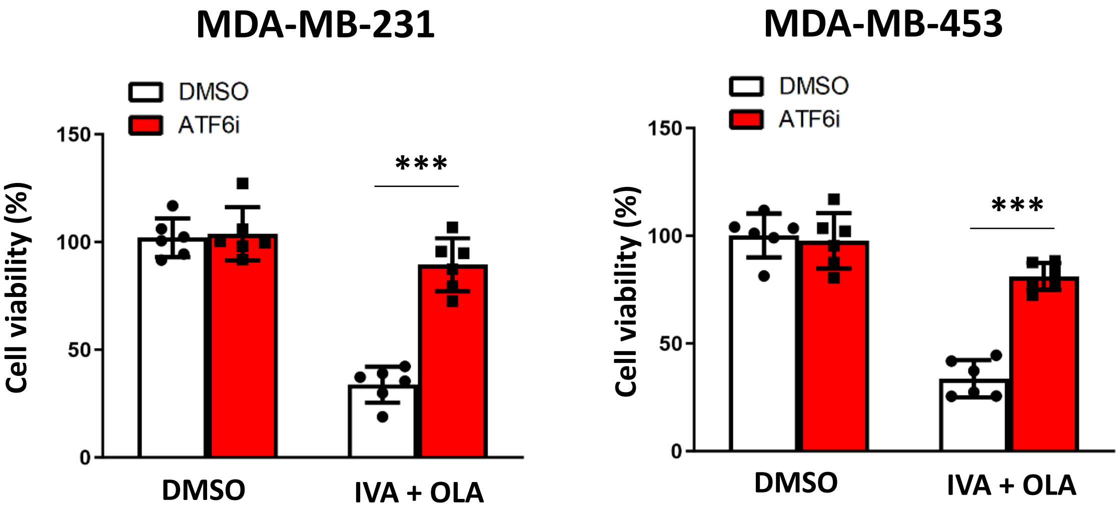

MDA-MB-231
DMSO
ATF6i
150
100
50
0
Cell viability (%)
***
DMSO
IVA + OLA
MDA-MB-453
DMSO
ATF6i
150
100
50
0
Cell viability (%)
***
DMSO
IVA + OLA

# Figure S12

MDA-MB-231

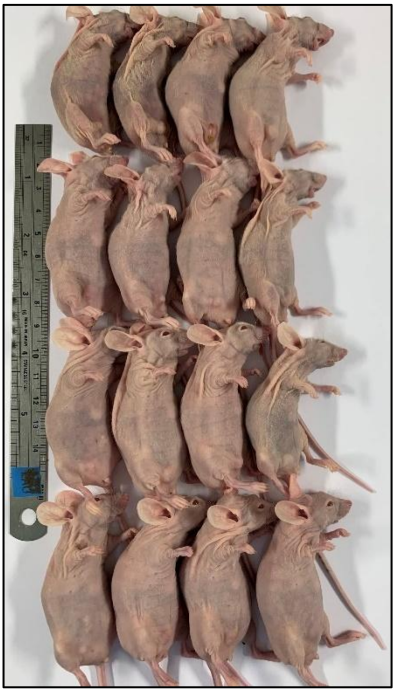

Saline

1mg/Kg COR

25mg/Kg LYN

1mg/Kg COR
+ 25mg/kg LYN

MDA-MB-453

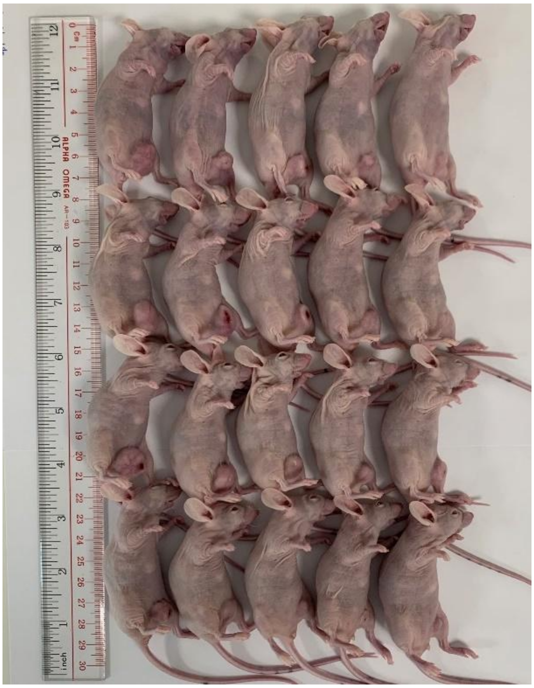

Saline

1mg/Kg COR

25mg/Kg LYN

1mg/Kg COR
+ 25mg/kg LYN

# Figure S13

**PDTX-5**

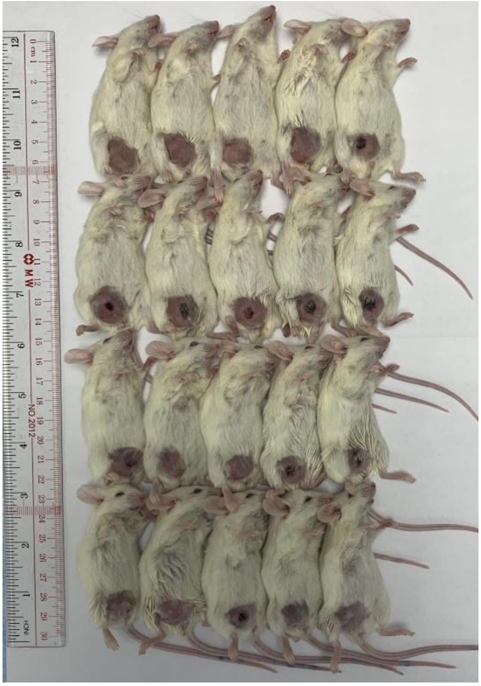

**Saline**

**2 mg/Kg COR**

**124 mg/Kg LYN**

**COR 2 mg/Kg COR + 124 mg/Kg LYN**

**PDTX-8**

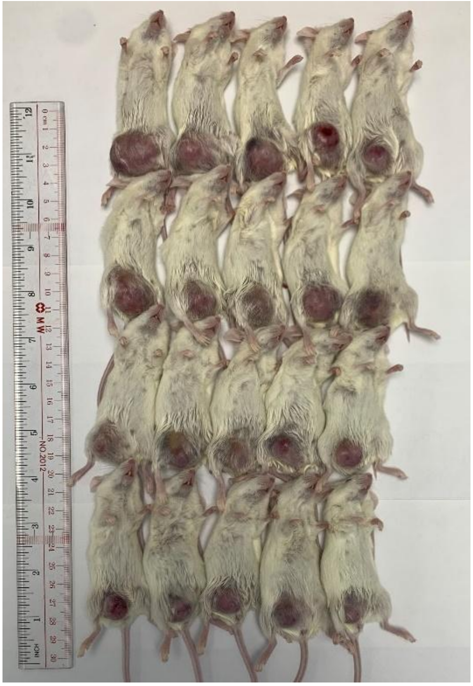

**Saline**

**2 mg/Kg COR**

**124 mg/Kg LYN**

**COR 2 mg/Kg COR + 124 mg/Kg LYN**
