# Supplementary material for: Ivabradine induces RAD51 degradation, potentiating PARP inhibitor efficacy in non-germline BRCA pathogenic variant triple-negative breast cancer
Source: J Transl Med. 2025 Aug 5;23:860. doi: 10.1186/s12967-025-06902-8 (PMC12323259; doi:10.1186/s12967-025-06902-8)
Supplement: Supplementary file 6 — Supplementary Material 6 [file 12967_2025_6902_MOESM6_ESM.docx]

**Supplementary figure legends**

**Figure S1 IVA did not affect the mRNA expression of *RAD51*, *BRCA1* and *BRCA2*.** A MDA-MB-231 and B MDA-MB-453 were treated with 0.1 µM of IVA for 72 hours. qPCR was employed to determine the gene expression. Actin was used as the internal control. Results were shown as mean ± SD from 3 independent experiments.

**Figure S2 A** the effect of tunicamycin on the expression of GRP78 and RAD51 in the cell lines. 0.1 µg/mL of tunicamycin (TUN) was employed to treat the cells. **B** the effect of IVA on GRP78 and RAD51 in the cell lines. 0.1 µM of IVA was used. Cells were harvested after 48 hours treatment. Western blot was performed. HSP90 was the loading control.

**Figure S3 IVA treatment alone did not significantly induce DNA damage.** MDA-MB-231 and MDA-MB-453 were treated with 0.1 µM of IVA for 72 hours. Comet assay was performed. 100 nuclei were analysed. Results were shown as mean ± SD. NT represents no chemical treatment.

**Figure S4 The representative trace from flow cytometry of the results from the TUNEL assay.** Supporting information for Fig. 4C. NT represents no chemical treatment.

**Figure S5 The effect of IVA and OLA in non-cancerous cells. A** The co-treatment of IVA and OLA on MCF10-A and HEK293 cells did not affect the expression of the indicated protein candidates. The cells were treated with 5 μM of OLA and 0.1 μM of IVA for 72 hours. Western blot was performed. GAPDH was the loading control. **B** The co-treatment of IVA and OLA did not affect the cell viability of MCF-10A and HEK293. The cells were treated with 5 μM of OLA and 0.1 μM of IVA for 72 hours. CCK8 was employed for assaying cell viability. Results were shown as mean ± SD from 4 independent experiments.

**Figure S6 Inhibition of A PERK and B IRE1α did not affect RAD51 expression.** MDA-MB-231 and MDA-MB-453 were treated with 0.1 μM of IVA for 72 hours. IRE1α was inhibited by 0.5 μM of Kira6 and PERK by 0.2 μM of GSK2606414. Western blot was performed. HSP90 was the loading control. The results were quantified and shown as mean ± SD from 3 independent experiments.

**Figure S7 The effect of IVA on the expression of ATF6 targeted F-box protein.** MDA-MB-231 and MDA-MB-453 were treated with 0.1 μM of IVA for 72 hours. qPCR was performed to determine the relative gene expression. Results were shown as mean ± SD from 6 independent experiments. Students’ t-test was employed. *** represents *P* < 0.001.

**Figure S8 Knockdown effect of *FBXO24***. MDA-MB-231 and MDA-MB-453 were treated with either 15 µM of non-targeting siRNA (siCtrl) or FBXO24 targeting siRNA (siFBXO24). Western blot was performed 48 hours post-transfection.

**Figure S9 4-PBA compromised the effect of the co-treatment on FBXO24 induction.** **A** 4-PBA treatment suppressed ATF6 from binding to *FBXO24* promoter in cells receiving the co-treatment. The cells were treated with 10 µM of 4-PBA, 5 μM of OLA and/or 0.1 μM of IVA for 72 hours. Chromatin immunoprecipitation (ChIP) was performed with anti-ATF6. qPCR was employed to determine the enrichment of -688 to -493 of *FBXO24* promoter in the elute. Results were shown as mean ± SD from 4 independent experiments. One-way ANOVA was employed. **B** 4-PBA compromised the effect of the co-treatment on *FBXO24* mRNA expression. Results were shown as mean ± SD from 4 independent experiments. One-way ANOVA was employed. **C** 4-PBA compromised the effect of the co-treatment on FBXO24 protein expression. HSP90 was the loading control. One-way ANOVA was employed. ** and *** represent *P* < 0.01 and *P* < 0.001, respectively.

**Figure S10 The effect of IVA, OLA and IVA + OLA on total ubiquitination levels in MDA-MB-231 and MDA-MB-453.** The cells were treated with 0.1 µM of IVA for 48 hours. Whole cell lysates were collected and subjected to western blot with anti-ubiquitin. Actin was the loading control. NT represents no chemical treatment.

**Figure S11 Inhibition of ATF6 compromised the efficacy of IVA and OLA co-treatment.** MDA-MB-231 and MDA-MB-453 were treated for 72 hours. 5 μM of ATF6 inhibitor Ceapin-A7, 0.1 μM of IVA and 5 μM of OLA were used. The cells were treated for 72 hours. Cell viability was performed using CCK8. Results were shown as mean ± SD from 6 independent experiments. Two-way ANOVA was employed. *** represents *P* < 0.001.

**Figure S12 Whole mice images of the nude mice treated with COR and LYN.** The xenografts were established from MDA-MB-231 and MDA-MB-453. The nude mice were treated with 1 mg/Kg of COR (clinical grade Ivabradine) and 25 mg/Kg of LYN (clinical grade Olaparib) via subcutaneous injection twice a week.

**Figure S13 Whole mice images of PDTX5 and PDTX8 treated with COR and LYN.** The mice were administered 2 mg/Kg of COR (clinical grade Ivabradine) and 124 mg/Kg of LYN (clinical grade Olaparib) via gavaged feeding daily.
