# Supplementary material for: Ivabradine induces RAD51 degradation, potentiating PARP inhibitor efficacy in non-germline BRCA pathogenic variant triple-negative breast cancer
Source: J Transl Med. 2025 Aug 5;23:860. doi: 10.1186/s12967-025-06902-8 (PMC12323259; doi:10.1186/s12967-025-06902-8)
Supplement: Supplementary file 7 — Supplementary Material 7 [file 12967_2025_6902_MOESM7_ESM.pdf]

Figure 1A

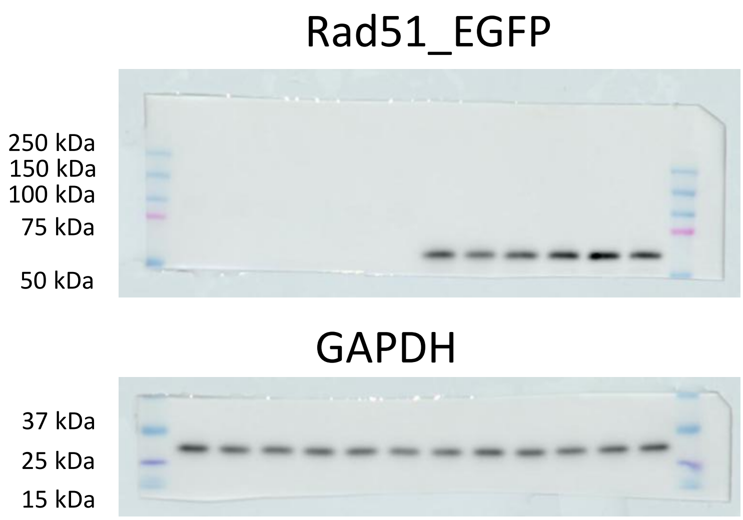
Rad51_EGFP
250 kDa
150 kDa
100 kDa
75 kDa
50 kDa
GAPDH
37 kDa
25 kDa
15 kDa

Figure 1E

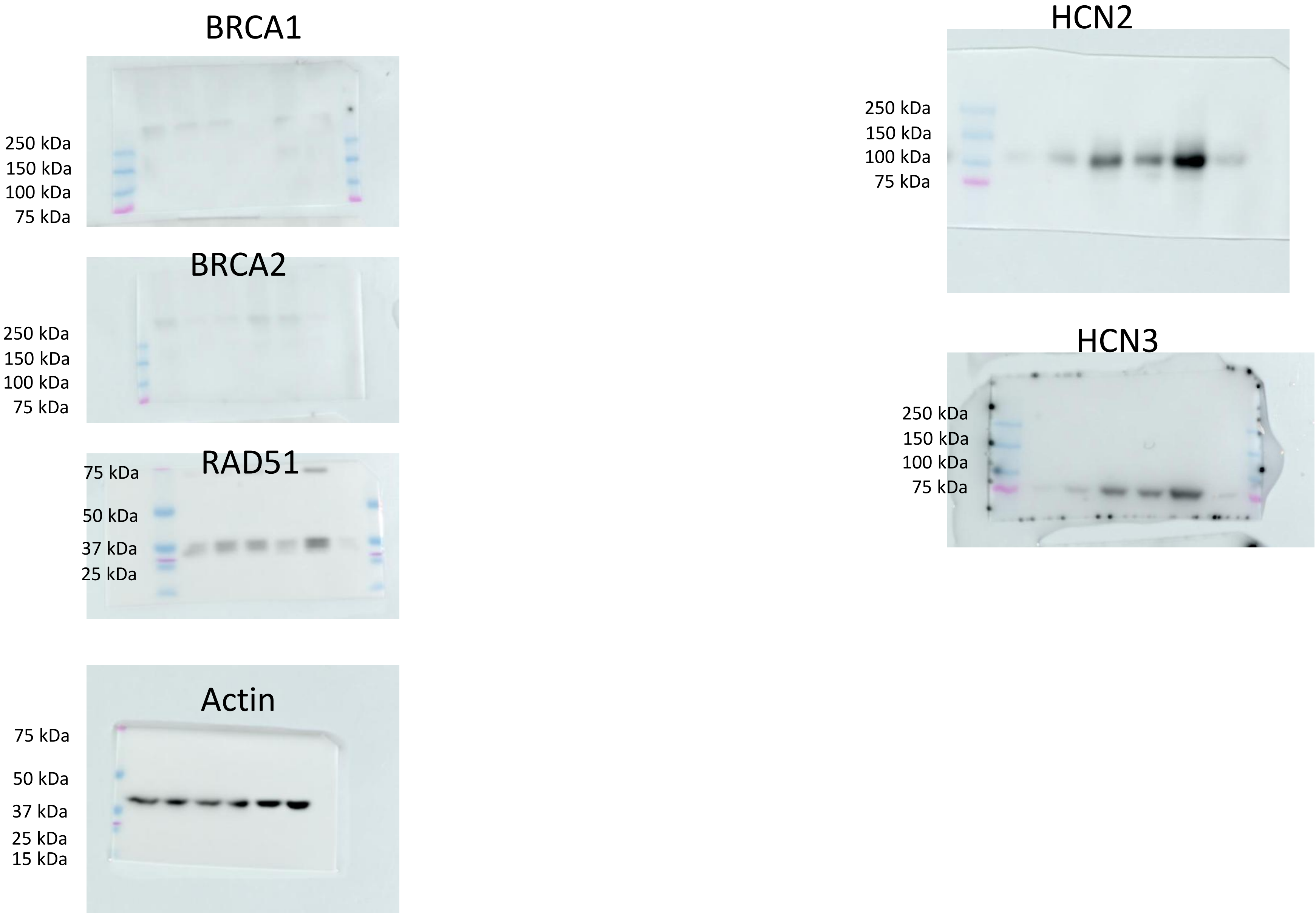
BRCA1
250 kDa
150 kDa
100 kDa
75 kDa
BRCA2
250 kDa
150 kDa
100 kDa
75 kDa
RAD51
75 kDa
50 kDa
37 kDa
25 kDa
Actin
75 kDa
50 kDa
37 kDa
25 kDa
15 kDa
HCN2
250 kDa
150 kDa
100 kDa
75 kDa
HCN3
250 kDa
150 kDa
100 kDa
75 kDa

Figure 3A

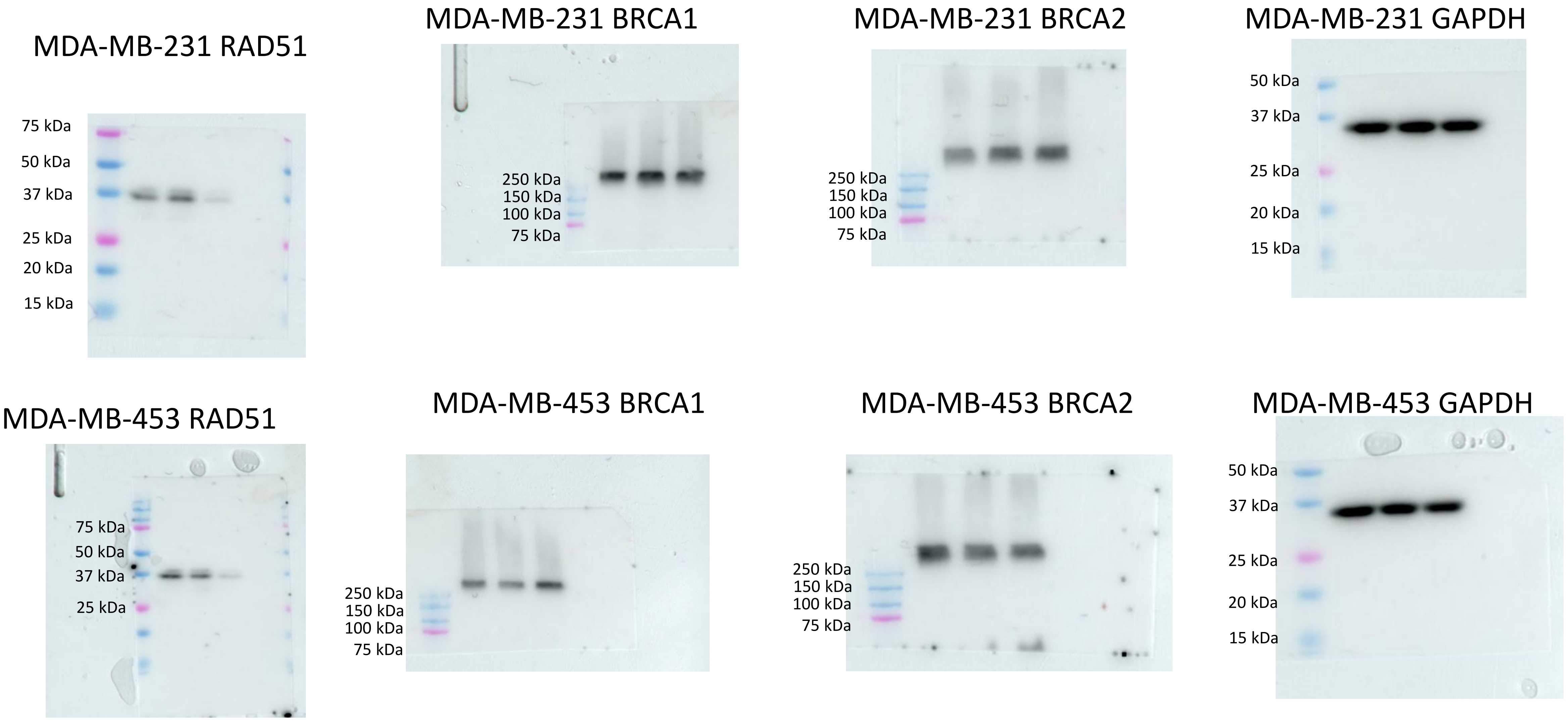
MDA-MB-231 RAD51
75 kDa
50 kDa
37 kDa
25 kDa
20 kDa
15 kDa
MDA-MB-231 BRCA1
250 kDa
150 kDa
100 kDa
75 kDa
MDA-MB-231 BRCA2
250 kDa
150 kDa
100 kDa
75 kDa
MDA-MB-231 GAPDH
50 kDa
37 kDa
25 kDa
20 kDa
15 kDa
MDA-MB-453 RAD51
75 kDa
50 kDa
37 kDa
25 kDa
MDA-MB-453 BRCA1
250 kDa
150 kDa
100 kDa
75 kDa
MDA-MB-453 BRCA2
250 kDa
150 kDa
100 kDa
75 kDa
MDA-MB-453 GAPDH
50 kDa
37 kDa
25 kDa
20 kDa
15 kDa

Figure 3B

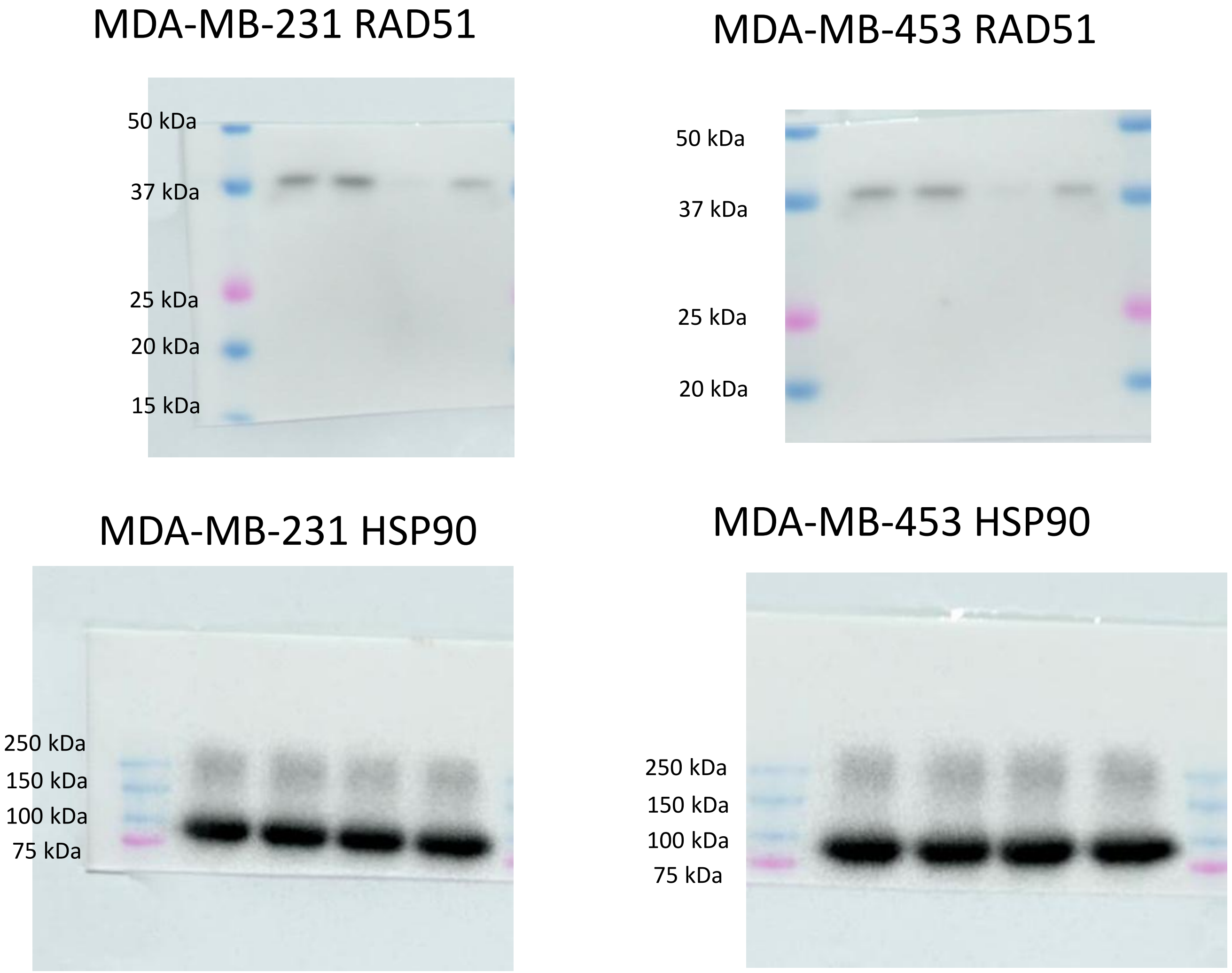
MDA-MB-231 RAD51
50 kDa
37 kDa
25 kDa
20 kDa
15 kDa
MDA-MB-453 RAD51
50 kDa
37 kDa
25 kDa
20 kDa
MDA-MB-231 HSP90
250 kDa
150 kDa
100 kDa
75 kDa
MDA-MB-453 HSP90
250 kDa
150 kDa
100 kDa
75 kDa

Figure 3D

MDA-MB-231 RAD51

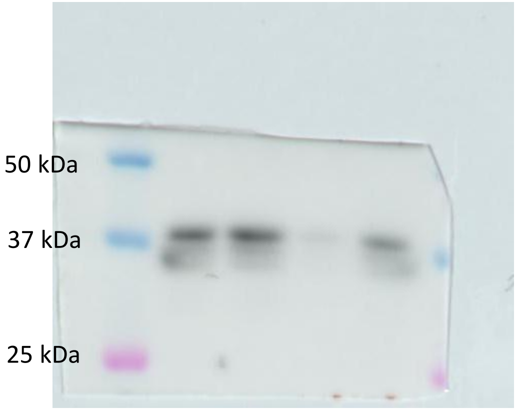

MDA-MB-453 RAD51

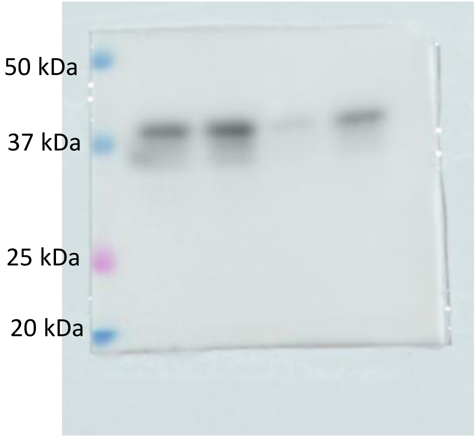

MDA-MB-231 HSP90

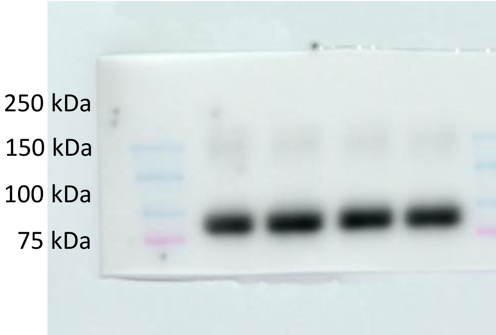

MDA-MB-453 HSP90

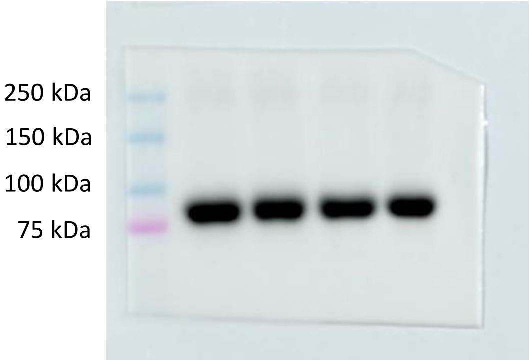

Figure 4D

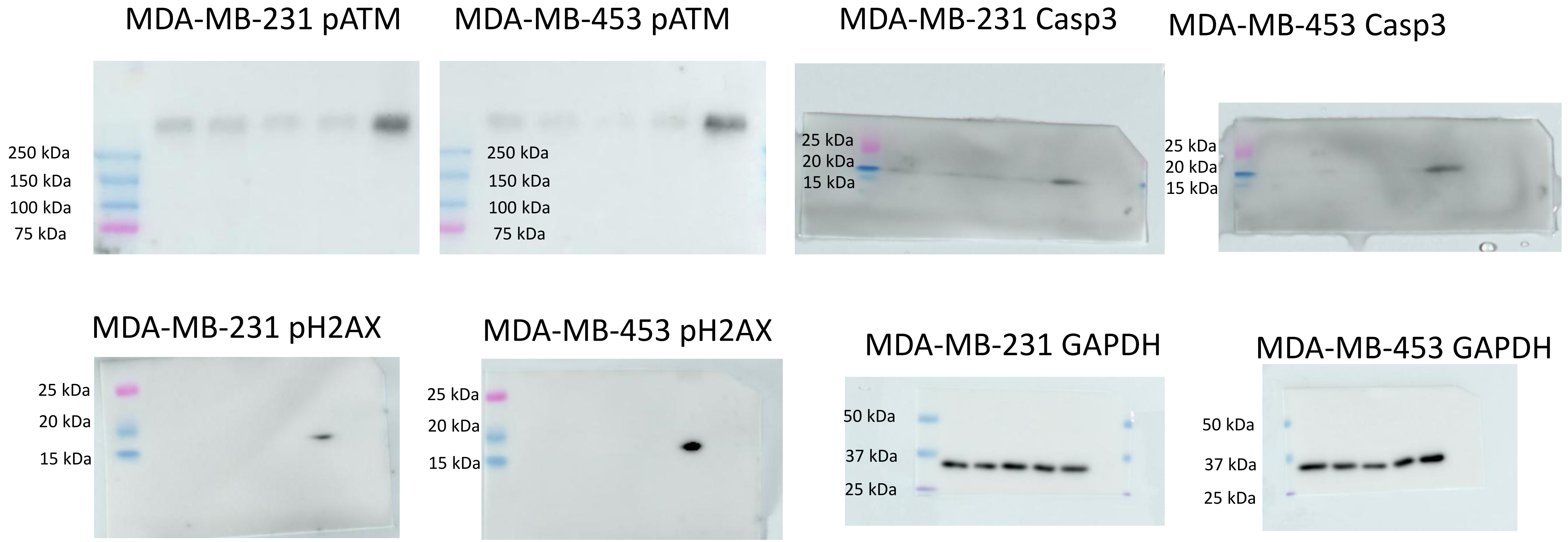

MDA-MB-231 pATM
MDA-MB-453 pATM
MDA-MB-231 Casp3
MDA-MB-453 Casp3
250 kDa
150 kDa
100 kDa
75 kDa
250 kDa
150 kDa
100 kDa
75 kDa
25 kDa
20 kDa
15 kDa
25 kDa
20 kDa
15 kDa
MDA-MB-231 pH2AX
MDA-MB-453 pH2AX
MDA-MB-231 GAPDH
MDA-MB-453 GAPDH
25 kDa
20 kDa
15 kDa
25 kDa
20 kDa
15 kDa
50 kDa
37 kDa
25 kDa
50 kDa
37 kDa
25 kDa

Figure 4G

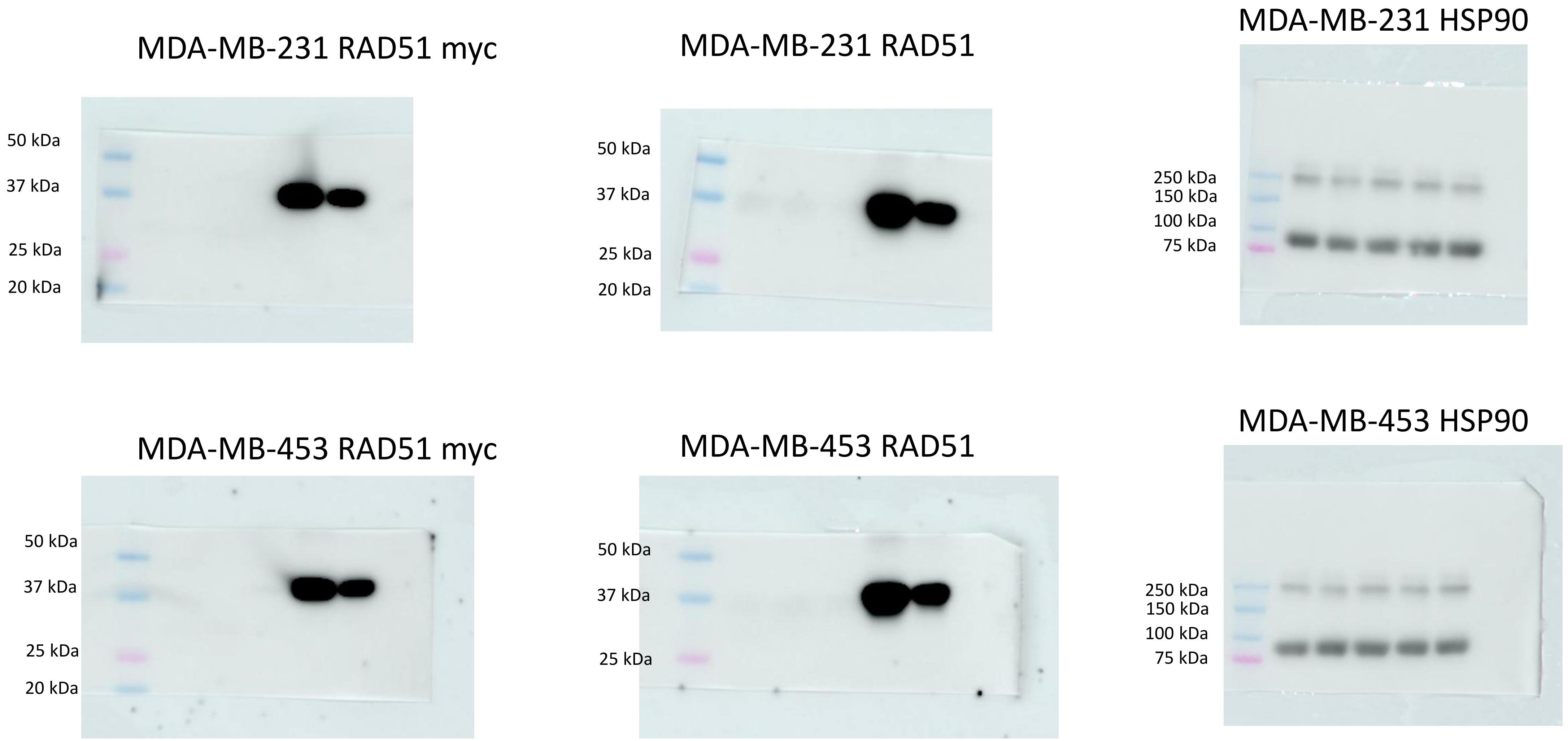
MDA-MB-231 RAD51 myc
50 kDa
37 kDa
25 kDa
20 kDa
MDA-MB-231 RAD51
50 kDa
37 kDa
25 kDa
20 kDa
MDA-MB-231 HSP90
250 kDa
150 kDa
100 kDa
75 kDa
MDA-MB-453 RAD51 myc
50 kDa
37 kDa
25 kDa
20 kDa
MDA-MB-453 RAD51
50 kDa
37 kDa
25 kDa
MDA-MB-453 HSP90
250 kDa
150 kDa
100 kDa
75 kDa

Figure 5A

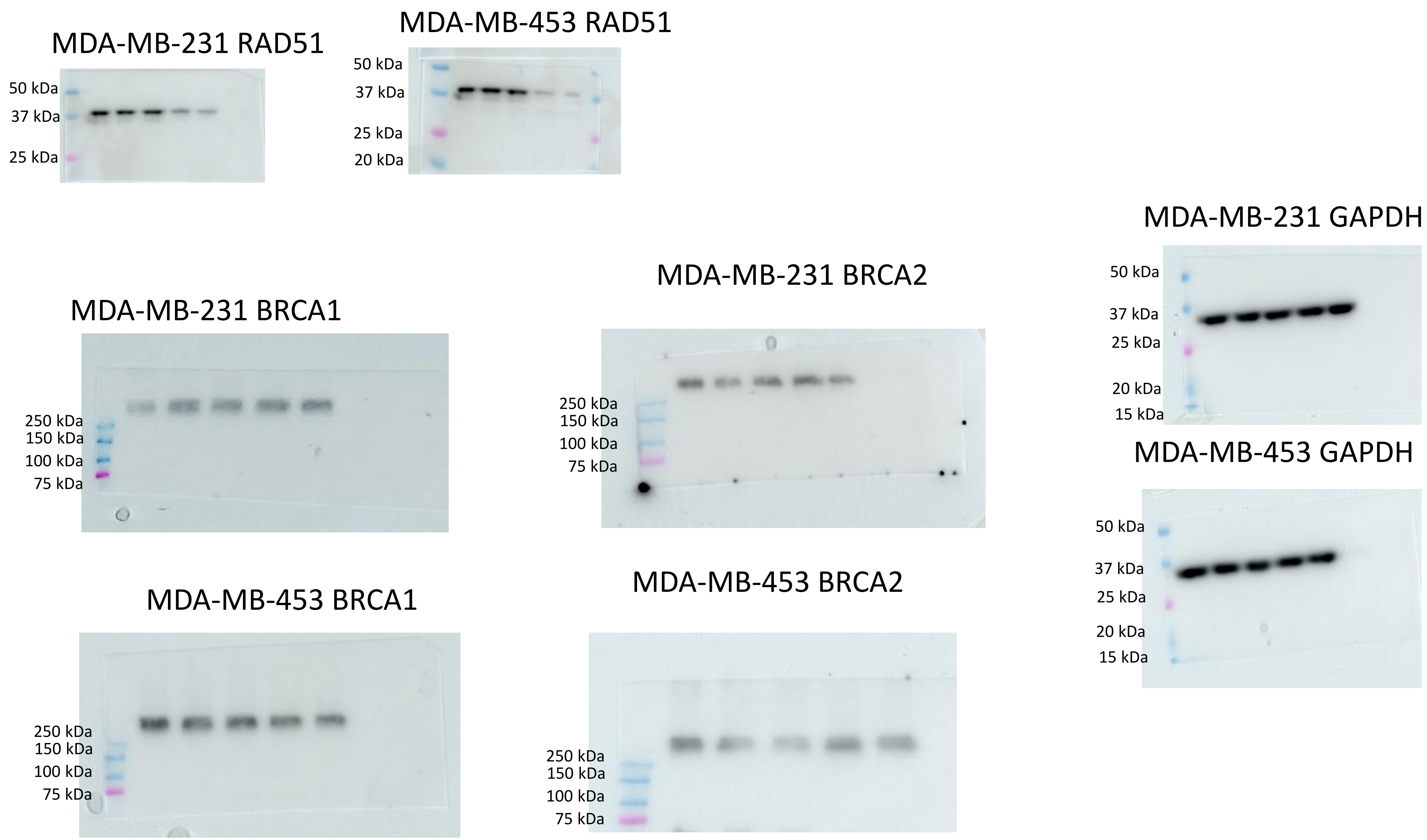
MDA-MB-231 RAD51
50 kDa
37 kDa
25 kDa
MDA-MB-453 RAD51
50 kDa
37 kDa
25 kDa
20 kDa
MDA-MB-231 GAPDH
50 kDa
37 kDa
25 kDa
20 kDa
15 kDa
MDA-MB-231 BRCA1
250 kDa
150 kDa
100 kDa
75 kDa
MDA-MB-231 BRCA2
250 kDa
150 kDa
100 kDa
75 kDa
MDA-MB-453 GAPDH
50 kDa
37 kDa
25 kDa
20 kDa
15 kDa
MDA-MB-453 BRCA1
250 kDa
150 kDa
100 kDa
75 kDa
MDA-MB-453 BRCA2
250 kDa
150 kDa
100 kDa
75 kDa

Figure 5B

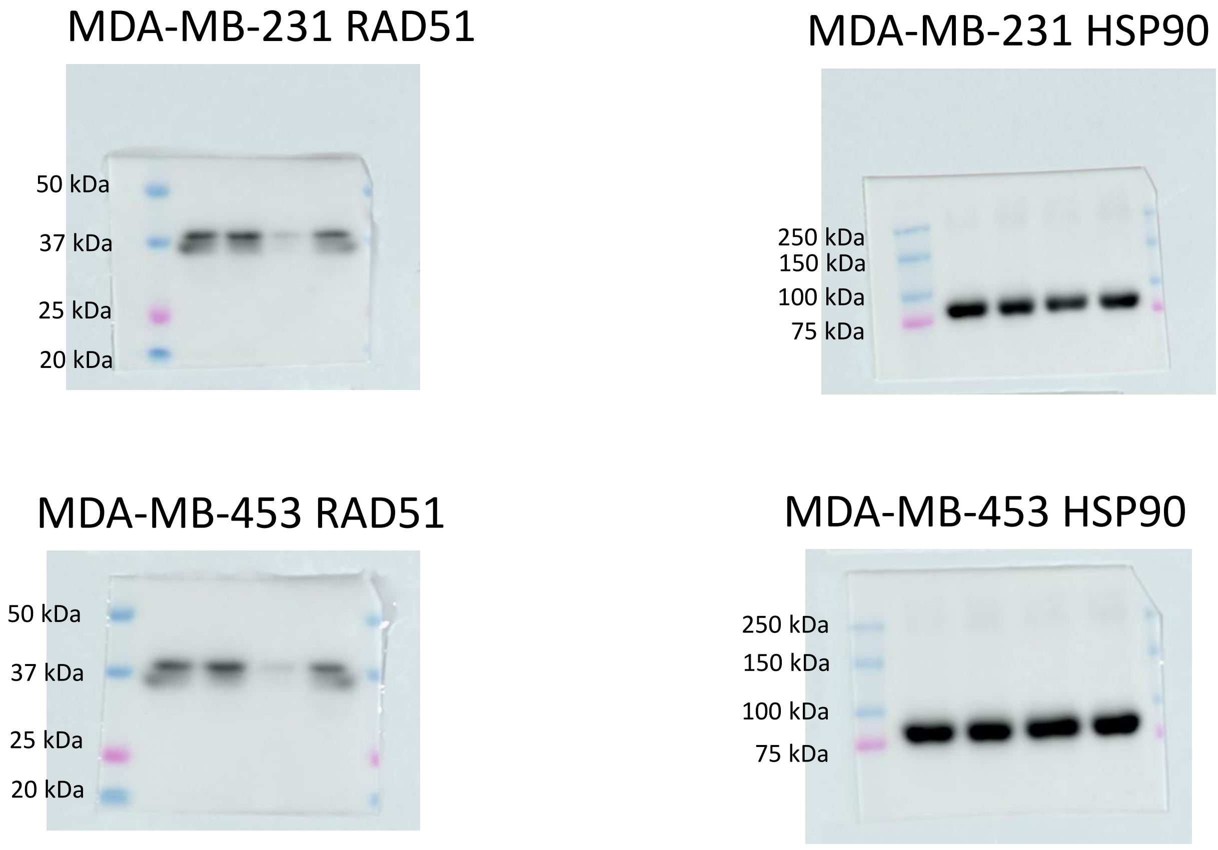

MDA-MB-231 RAD51
50 kDa
37 kDa
25 kDa
20 kDa
MDA-MB-231 HSP90
250 kDa
150 kDa
100 kDa
75 kDa
MDA-MB-453 RAD51
50 kDa
37 kDa
25 kDa
20 kDa
MDA-MB-453 HSP90
250 kDa
150 kDa
100 kDa
75 kDa

Figure 5E

MDA-MB-231 FBXO24

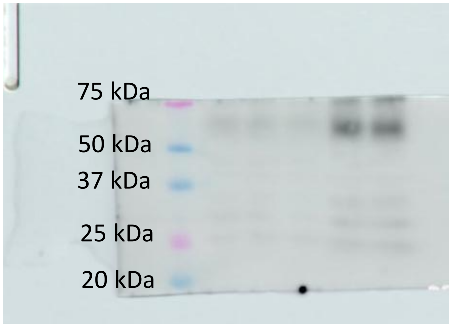

MDA-MB-231 HSP90

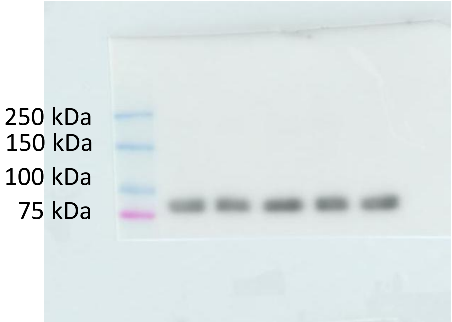

MDA-MB-453 FBXO24

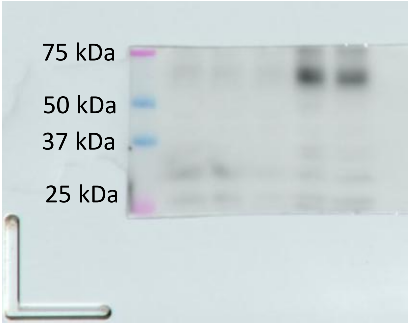

MDA-MB-453 HSP90

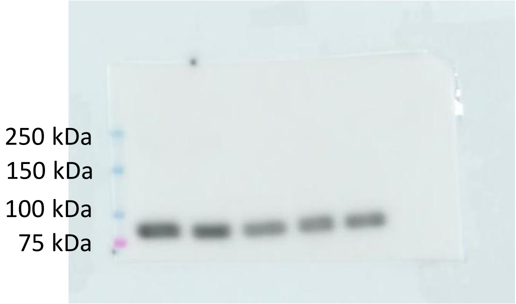

Figure 5F

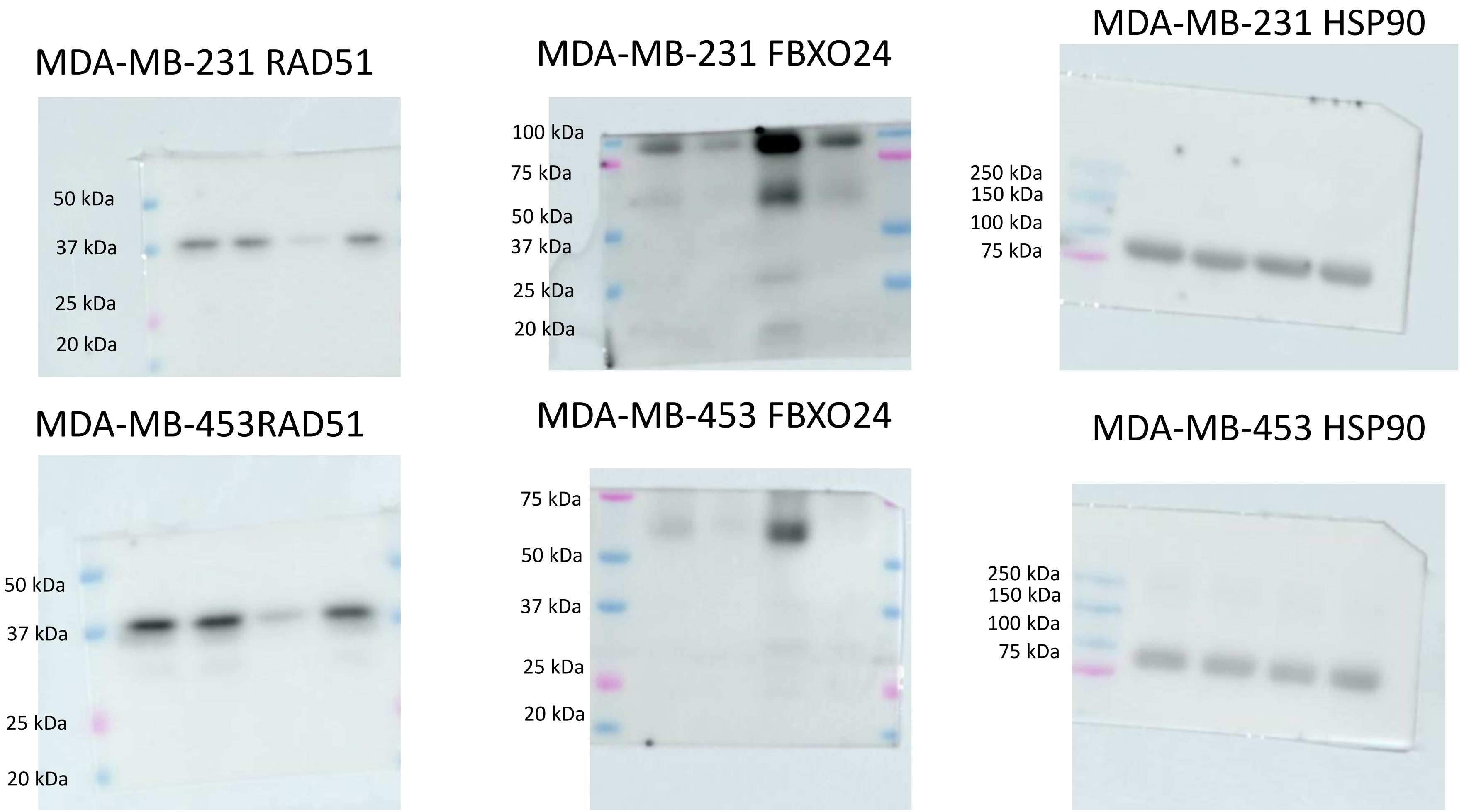
MDA-MB-231 RAD51
50 kDa
37 kDa
25 kDa
20 kDa
MDA-MB-231 FBXO24
100 kDa
75 kDa
50 kDa
37 kDa
25 kDa
20 kDa
MDA-MB-231 HSP90
250 kDa
150 kDa
100 kDa
75 kDa
MDA-MB-453RAD51
50 kDa
37 kDa
25 kDa
20 kDa
MDA-MB-453 FBXO24
75 kDa
50 kDa
37 kDa
25 kDa
20 kDa
MDA-MB-453 HSP90
250 kDa
150 kDa
100 kDa
75 kDa

Figure 6A

MDA-MB-231 FBXO24 elute

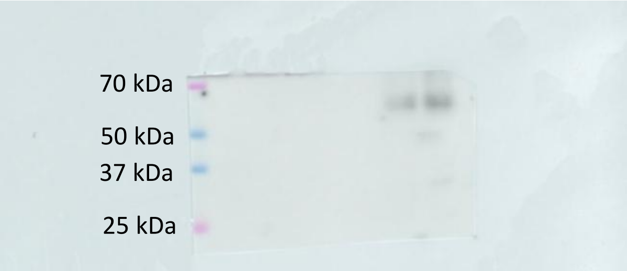

MDA-MB-231 RAD51myc elute

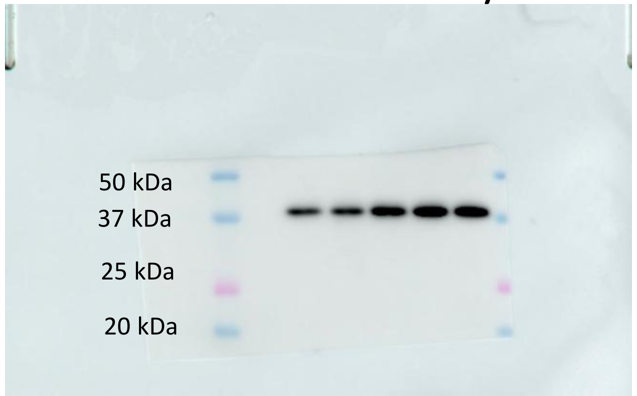

MDA-MB-453 FBXO24 elute

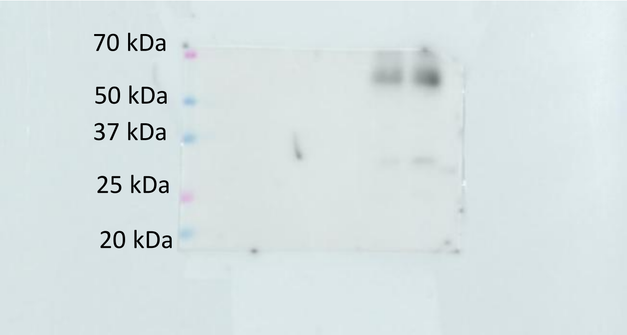

MDA-MB-453 RAD51myc elute

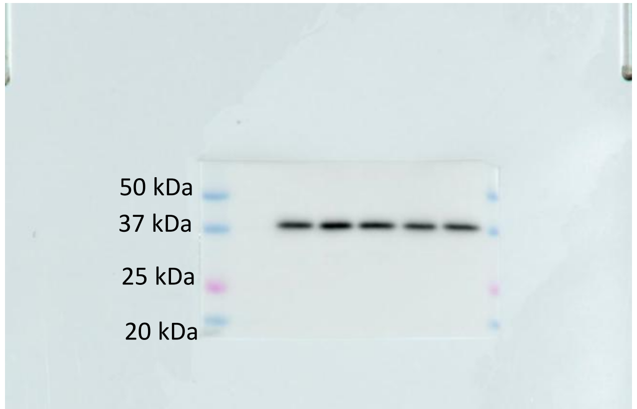

Figure 6B

MDA-MB-231 RAD51myc elute

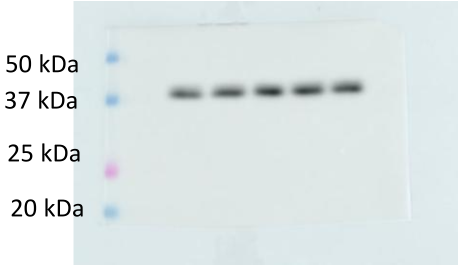

MDA-MB-231 UB-K48 elute

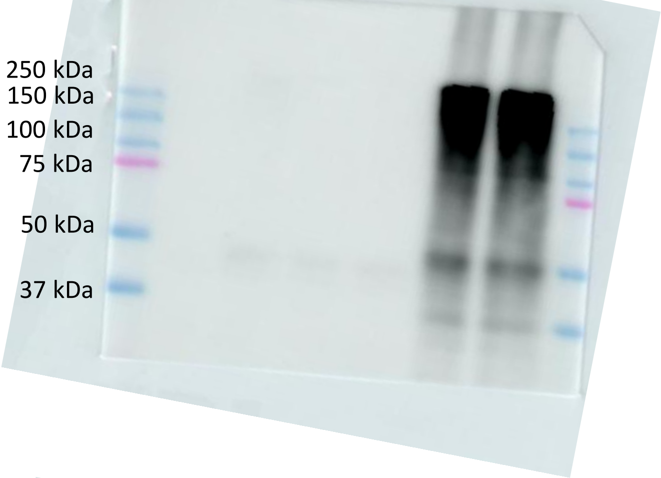

MDA-MB-453 RAD51myc elute

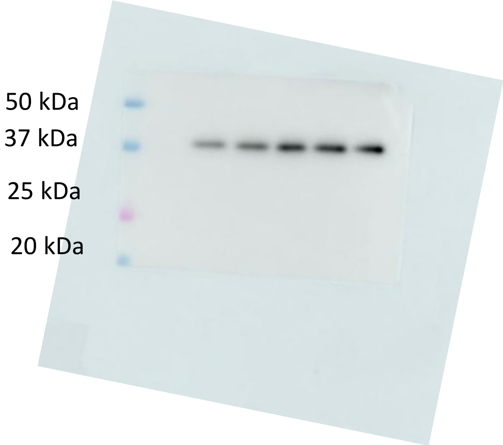

MDA-MB-453 UB-K48 elute

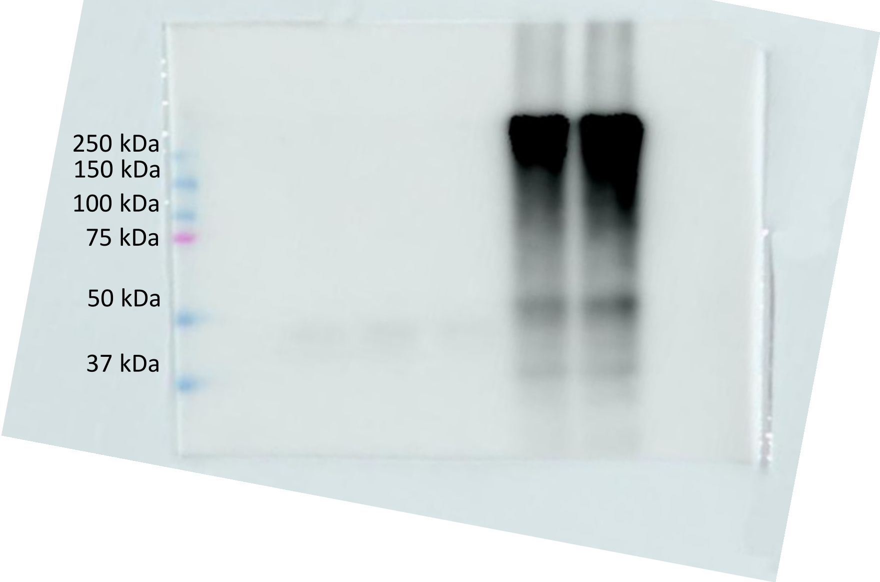

Figure 6C

MDA-MB-231 RAD51myc elute

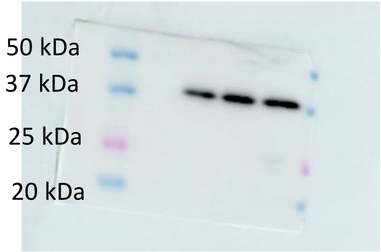

MDA-MB-231 UB-K48 elute

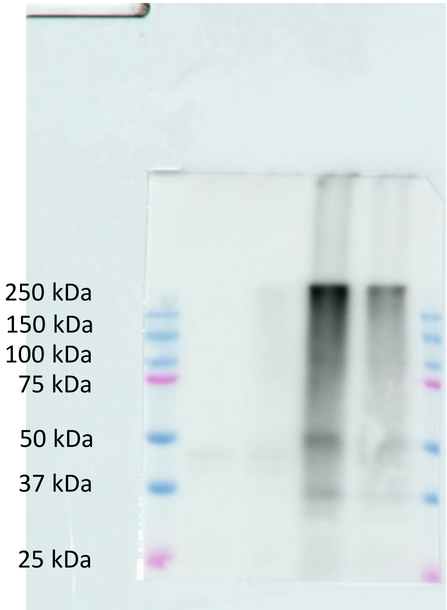

MDA-MB-453 RAD51myc elute

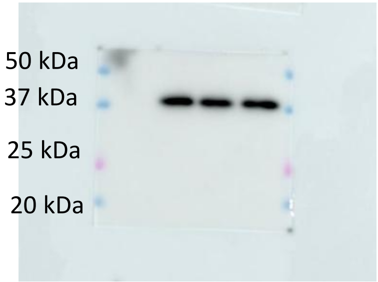

MDA-MB-453 UB-K48 elute

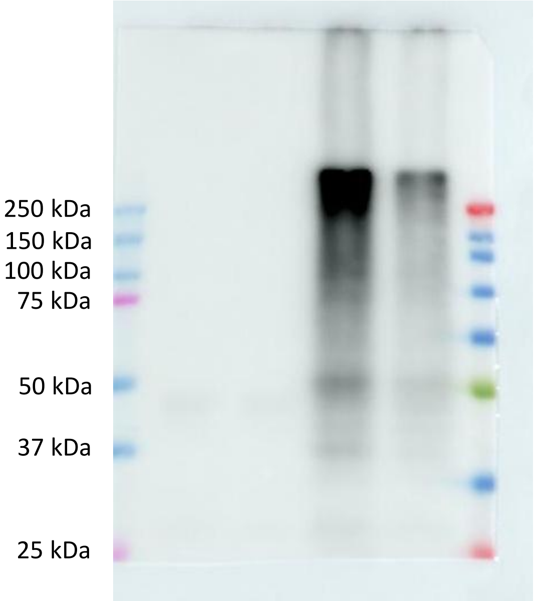

Figure 7C

BRCA1 MDA-MB-231 xenograft

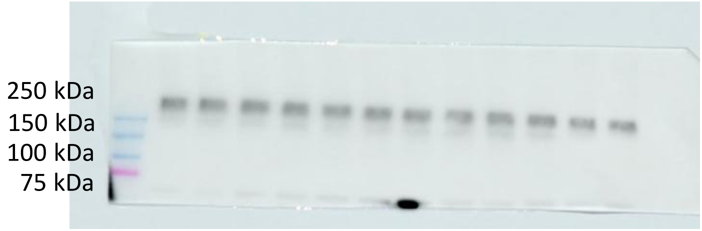

BRCA1 MDA-MB-453 xenograft

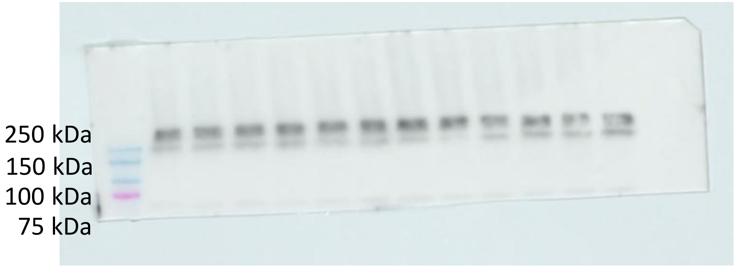

BRCA2 MDA-MB-231 xenograft

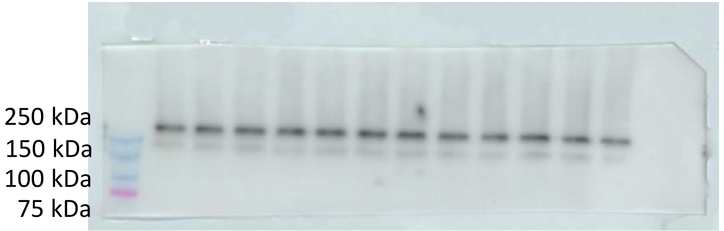

BRCA2 MDA-MB-453 xenograft

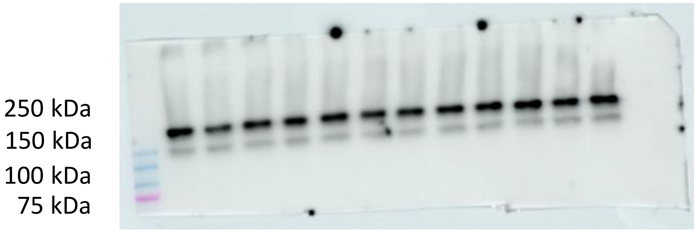

RAD51 MDA-MB-231 xenograft

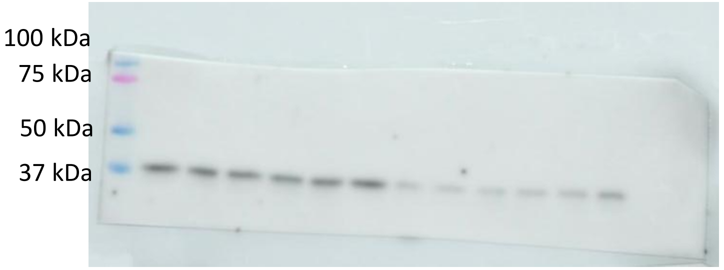

RAD51 MDA-MB-453 xenograft

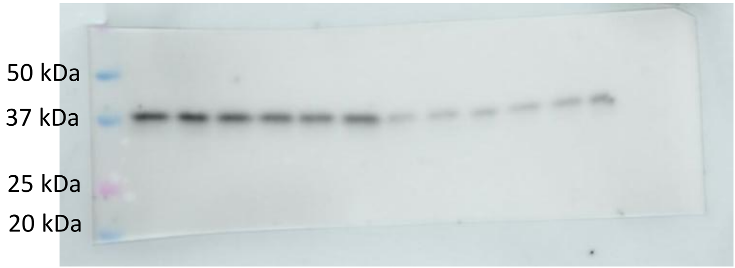

FBXO24 MDA-MB-231 xenograft

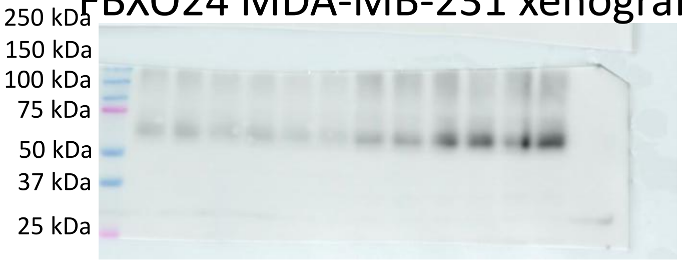

FBXO24 MDA-MB-453 xenograft

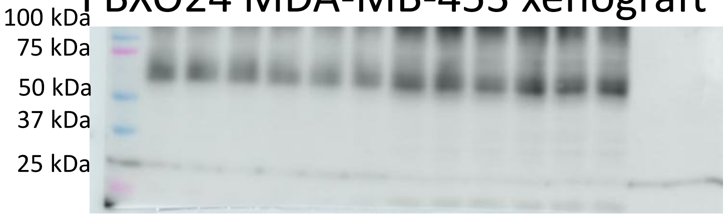

GAPDH MDA-MB-231 xenograft

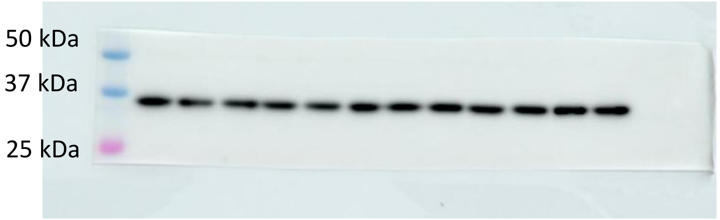

GAPDH MDA-MB-453 xenograft

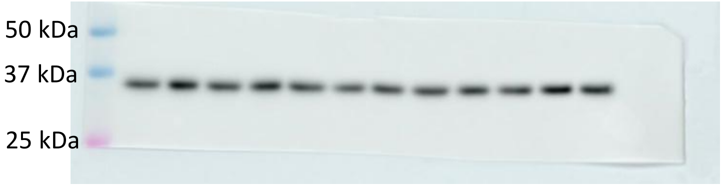

P-ATM MDA-MB-231 xenograft

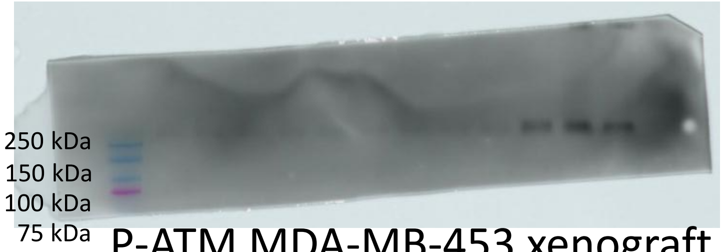

P-ATM MDA-MB-453 xenograft

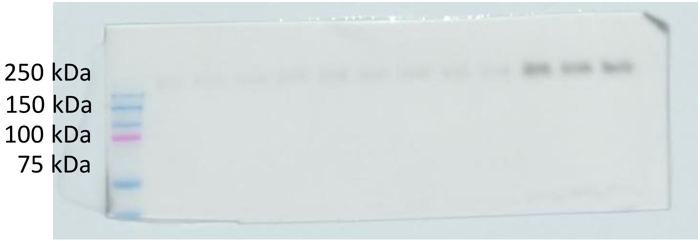

Figure 8C

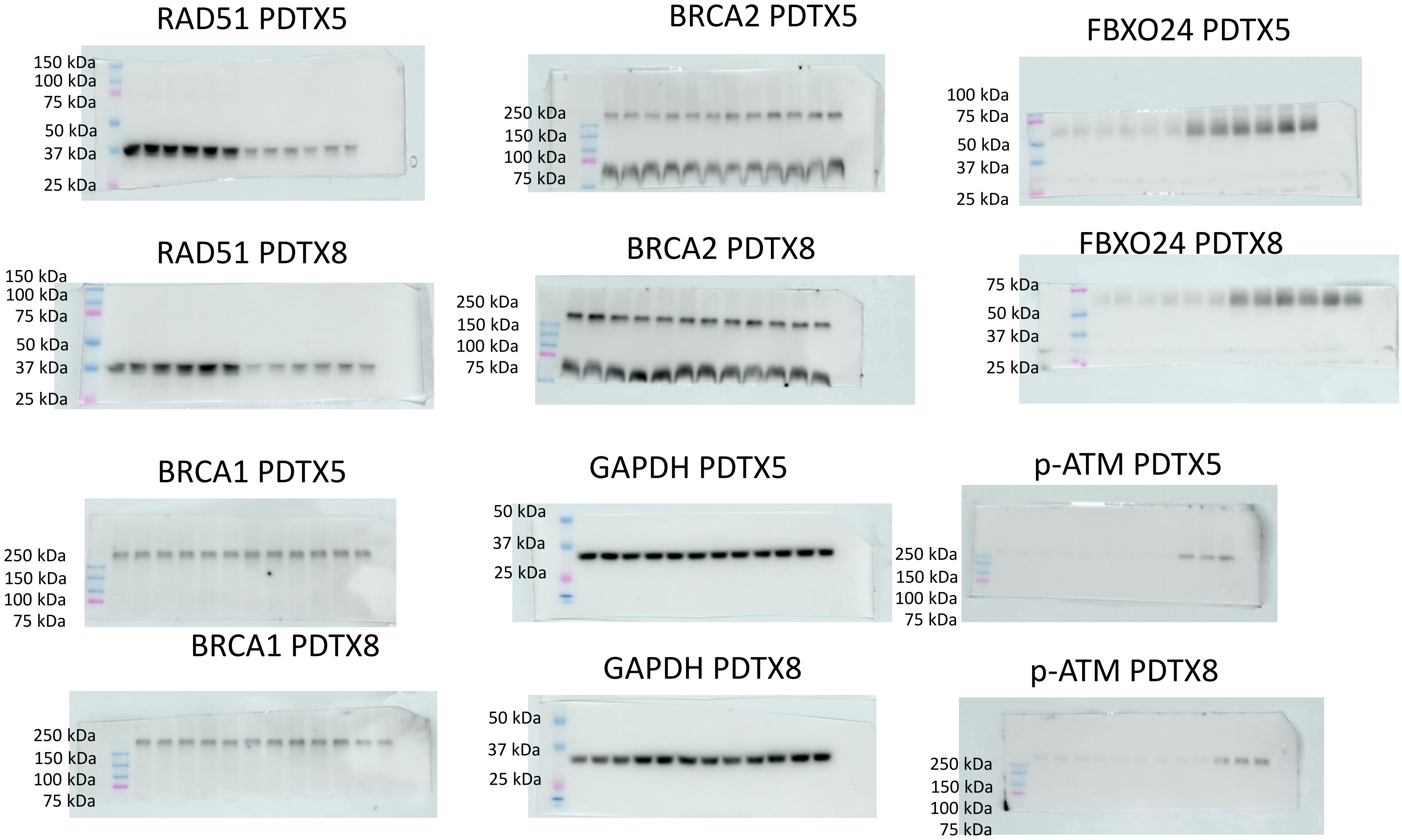
RAD51 PDTX5
150 kDa
100 kDa
75 kDa
50 kDa
37 kDa
25 kDa
BRCA2 PDTX5
250 kDa
150 kDa
100 kDa
75 kDa
FBXO24 PDTX5
100 kDa
75 kDa
50 kDa
37 kDa
25 kDa
RAD51 PDTX8
150 kDa
100 kDa
75 kDa
50 kDa
37 kDa
25 kDa
BRCA2 PDTX8
250 kDa
150 kDa
100 kDa
75 kDa
FBXO24 PDTX8
75 kDa
50 kDa
37 kDa
25 kDa
BRCA1 PDTX5
250 kDa
150 kDa
100 kDa
75 kDa
GAPDH PDTX5
50 kDa
37 kDa
25 kDa
p-ATM PDTX5
250 kDa
150 kDa
100 kDa
75 kDa
BRCA1 PDTX8
250 kDa
150 kDa
100 kDa
75 kDa
GAPDH PDTX8
50 kDa
37 kDa
25 kDa
p-ATM PDTX8
250 kDa
150 kDa
100 kDa
75 kDa

Supplementary Figures

Figure S2A

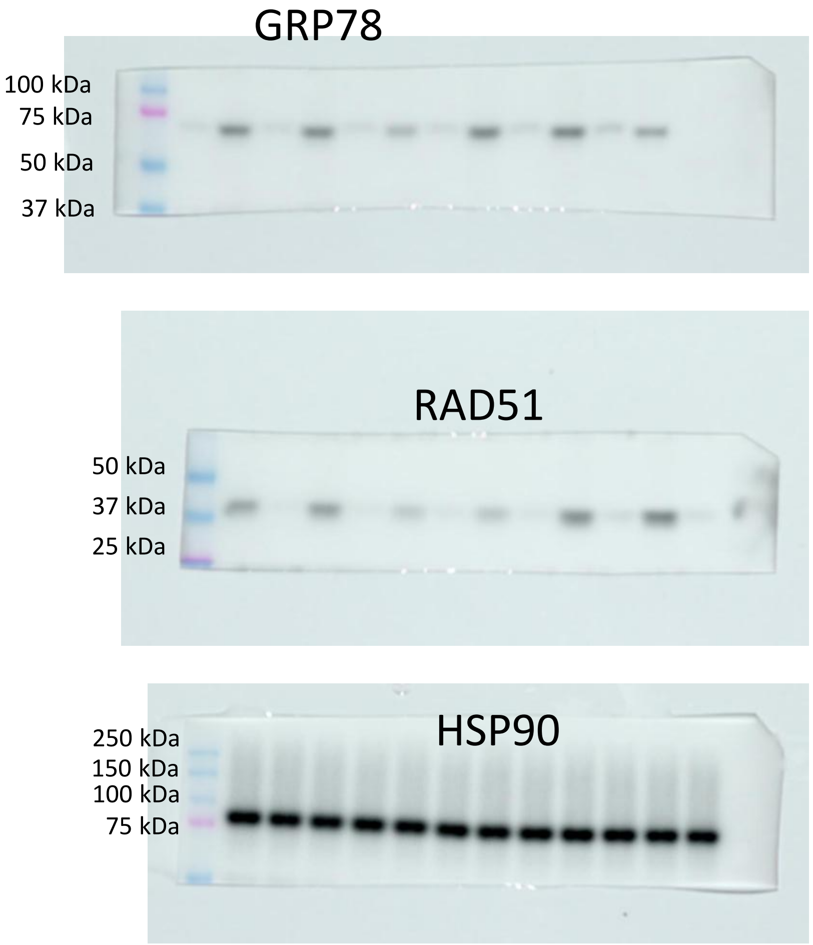

Figure S2B

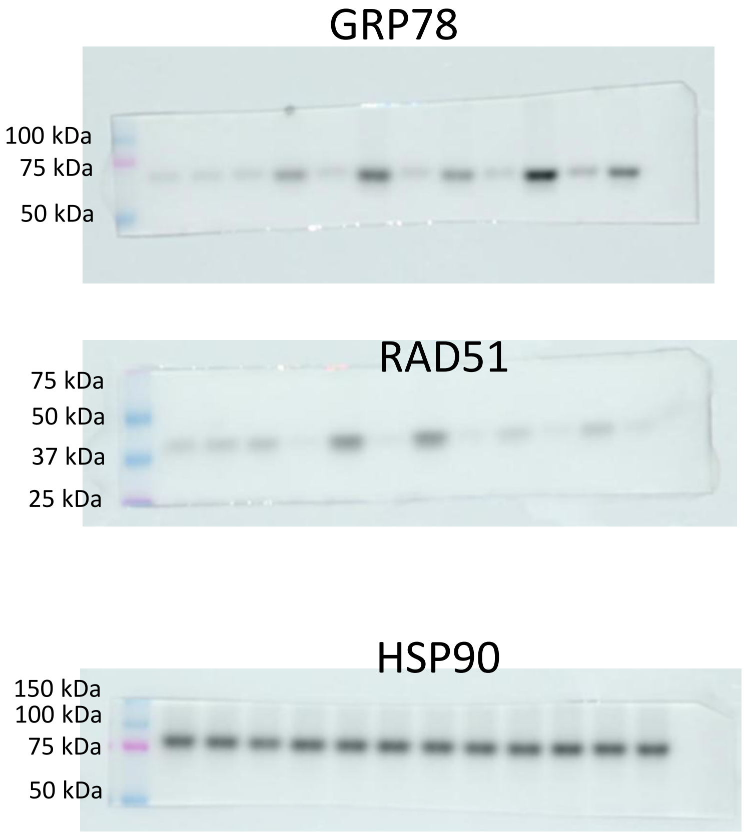

Figure S5

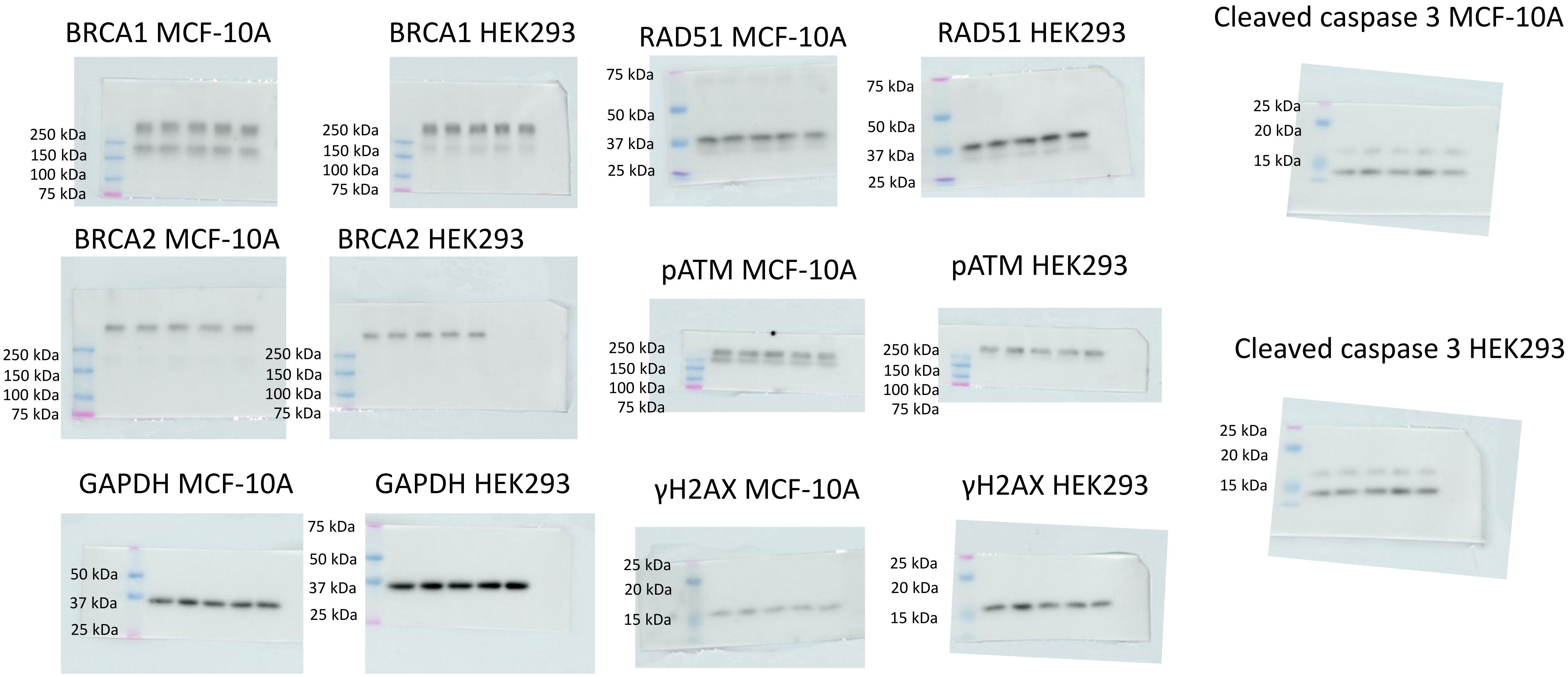
BRCA1 MCF-10A
250 kDa
150 kDa
100 kDa
75 kDa
BRCA1 HEK293
250 kDa
150 kDa
100 kDa
75 kDa
RAD51 MCF-10A
75 kDa
50 kDa
37 kDa
25 kDa
RAD51 HEK293
75 kDa
50 kDa
37 kDa
25 kDa
Cleaved caspase 3 MCF-10A
25 kDa
20 kDa
15 kDa
BRCA2 MCF-10A
250 kDa
150 kDa
100 kDa
75 kDa
BRCA2 HEK293
250 kDa
150 kDa
100 kDa
75 kDa
pATM MCF-10A
250 kDa
150 kDa
100 kDa
75 kDa
pATM HEK293
250 kDa
150 kDa
100 kDa
75 kDa
Cleaved caspase 3 HEK293
25 kDa
20 kDa
15 kDa
GAPDH MCF-10A
50 kDa
37 kDa
25 kDa
GAPDH HEK293
75 kDa
50 kDa
37 kDa
25 kDa
γH2AX MCF-10A
25 kDa
20 kDa
15 kDa
γH2AX HEK293
25 kDa
20 kDa
15 kDa

Figure S6

MDA-MB-231 IRE1ai RAD51

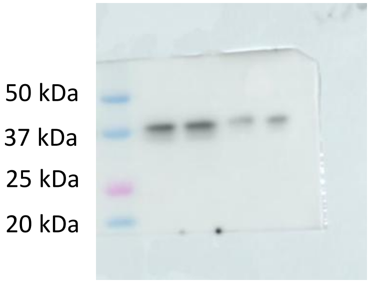

MDA-MB-231 IRE1ai HSP90

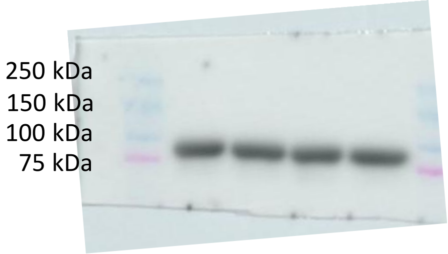

MDA-MB-231 PERKi RAD51

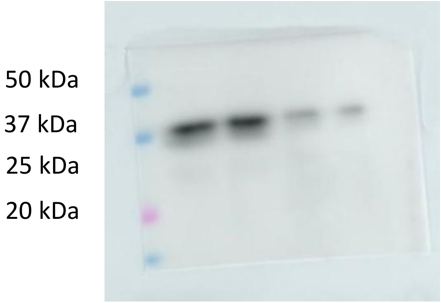

MDA-MB-231 PERKi HSP90

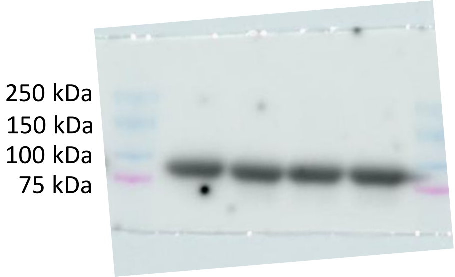

MDA-MB-453 IRE1ai RAD51

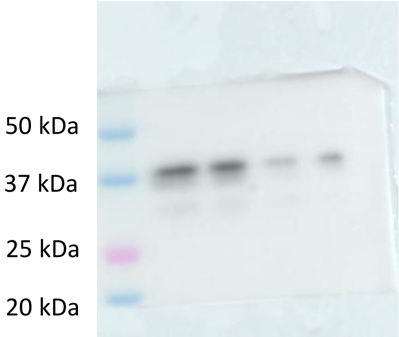

MDA-MB-453 IRE1ai HSP90

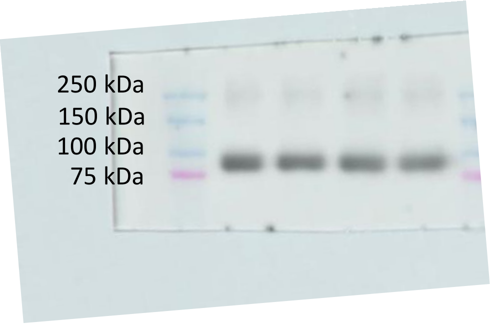

MDA-MB-453 PERKi RAD51

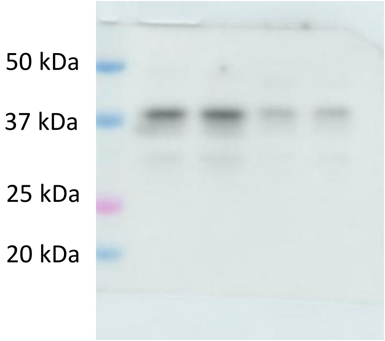

MDA-MB-453 PERKi HSP90

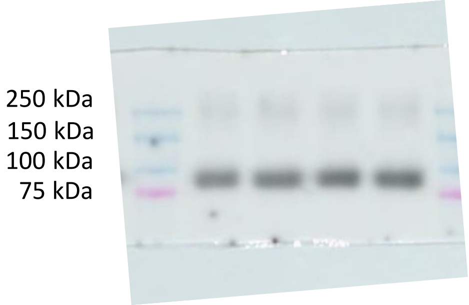

Figure S8

MDA-MB-231 FBXO24

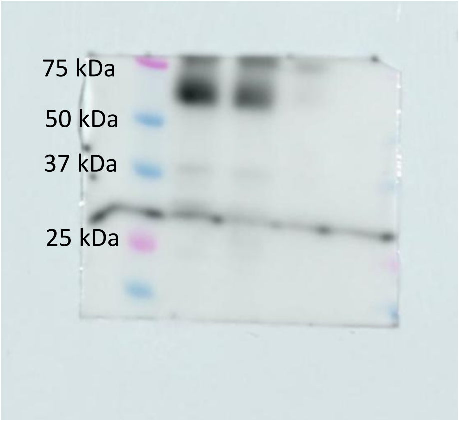

MDA-MB-453 FBXO24

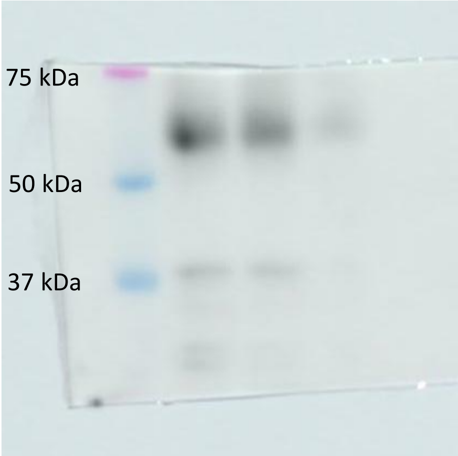

MDA-MB-231 HSP90

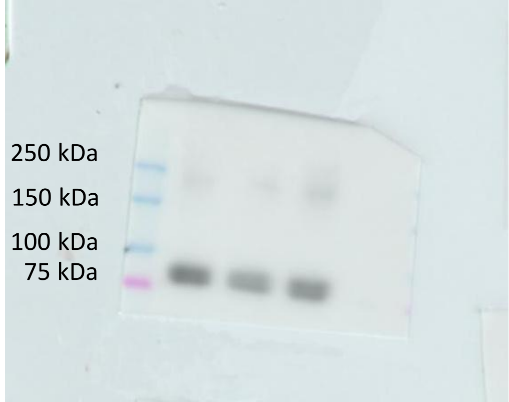

MDA-MB-453 HSP90

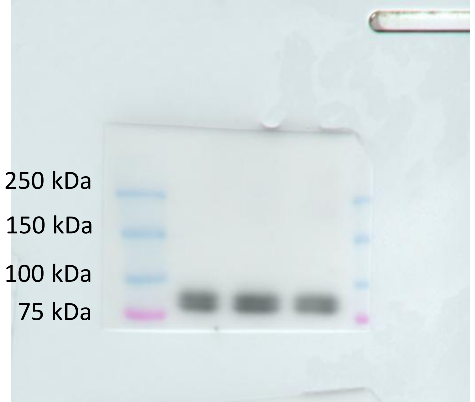

Figure S9C

MDA-MB-231 FBXO24

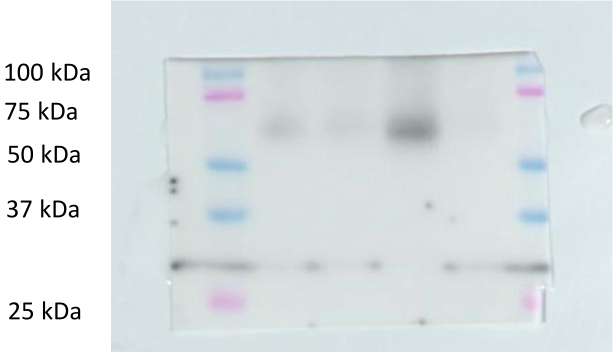

MDA-MB-453 FBXO24

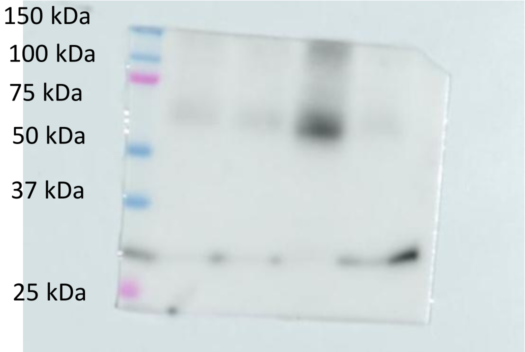

MDA-MB-231 HSP90

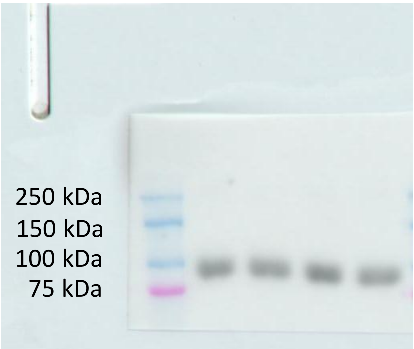

MDA-MB-453 HSP90

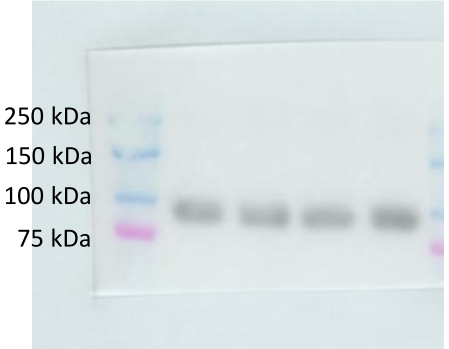

Figure S10

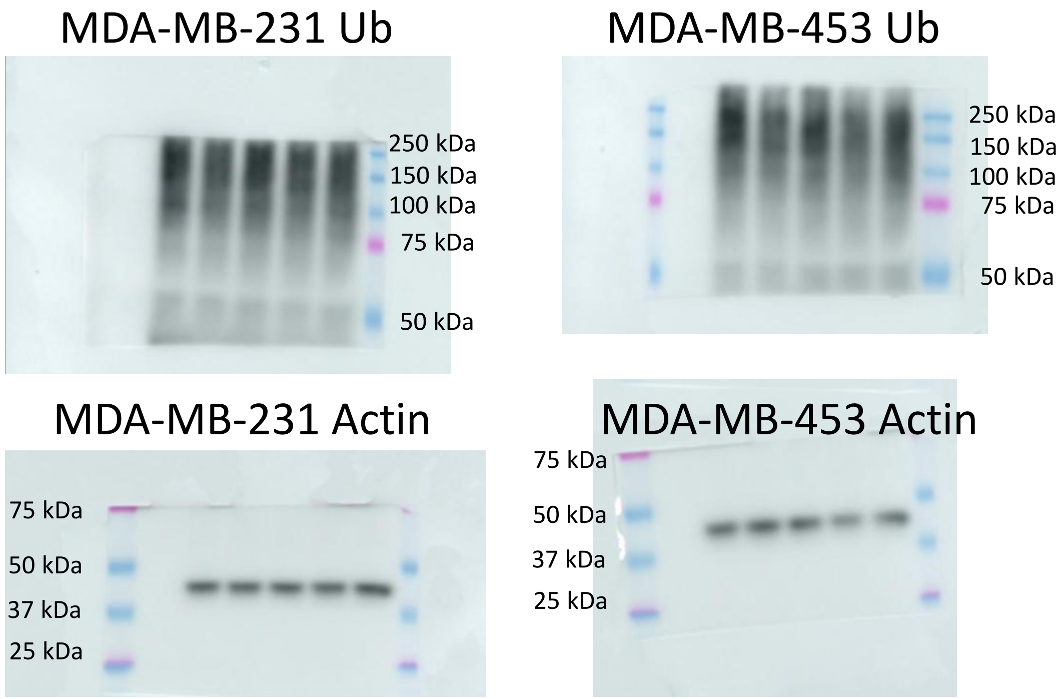
MDA-MB-231 Ub
250 kDa
150 kDa
100 kDa
75 kDa
50 kDa
MDA-MB-453 Ub
250 kDa
150 kDa
100 kDa
75 kDa
50 kDa
MDA-MB-231 Actin
75 kDa
50 kDa
37 kDa
25 kDa
MDA-MB-453 Actin
75 kDa
50 kDa
37 kDa
25 kDa
